# Roles of the Gac-Rsm pathway in the regulation of phenazine biosynthesis in *Pseudomonas chlororaphis* 30-84

**DOI:** 10.1002/mbo3.90

**Published:** 2013-04-21

**Authors:** Dongping Wang, Sung-Hee Lee, Candace Seeve, Jun Myoung Yu, Leland S Pierson, Elizabeth A Pierson

**Affiliations:** 1 Department of Plant Pathology and Microbiology, Texas A&M UniversityCollege Station, Texas, 77843-2133; 2 Horticultural Research Division, Chungcheongbuk-Do Agricultural Research & Extension ServicesOchang-eup, Chungcheongbuk-Do, 363-883, South Korea; 3 Department of Horticultural Sciences, Texas A&M UniversityCollege Station, Texas, 77843-2133; 4 Department of Biology, Baylor UniversityWaco, Texas, 76798

**Keywords:** Biological control, Gac, phenazine, posttranscriptional regulation, *Pseudomonas*, two-component signal transduction

## Abstract

The GacS/GacA two-component regulatory system activates the production of secondary metabolites including phenazines crucial for biological control activity in *Pseudomonas chlororaphis* 30-84. To better understand the role of the Gac system on phenazine regulation, transcriptomic analyses were conducted by comparing the wild-type strain to a *gacA* mutant. RNA-seq analysis identified 771 genes under GacA control, including many novel genes. Consistent with previous findings, phenazine biosynthetic genes were significantly downregulated in a *gacA* mutant. The transcript abundances of phenazine regulatory genes such as *phzI*, *phzR*, *iopA*, *iopB*, *rpoS*, and *pip* also were reduced. Moreover, the transcript abundance of three noncoding RNAs (ncRNAs) including *rsmX*, *rsmY*, and *rsmZ* was significantly decreased by *gacA* mutation consistent with the presence of consensus GacA-binding sites associated with their promoters. Our results also demonstrated that constitutive expression of *rsmZ* from a non-*gac* regulated promoter resulted in complete restoration of *N*-acyl-homoserine lactone (AHL) and phenazine production as well as the expression of other *gac*-dependent secondary metabolites in *gac* mutants. The role of RsmA and RsmE in phenazine production also was investigated. Overexpression of *rsmE*, but not *rsmA*, resulted in decreased AHL and phenazine production in *P. chlororaphis*, and only a mutation in *rsmE* bypassed the requirement for GacA in phenazine gene expression. In contrast, constitutive expression of the *phzI*/*phzR* quorum sensing system did not rescue phenazine production in the *gacA* mutant, indicating the direct posttranscriptional control by Gac on the phenazine biosynthetic genes. On the basis of these results, we propose a model to illustrate the hierarchic role of phenazine regulators modulated by Gac in the control of phenazine production. The transcriptomic analysis also was used to identify additional genes regulated by GacA that may contribute to the biological control capability of strain 30-84.

## Introduction

The GacS/GacA two-component signal transduction system (TCST) is highly conserved among Gram-negative bacteria, including many beneficial biological control and plant pathogenic bacteria (Heeb and Haas 2001). The Gac system was originally identified as a global activator of antibiotic and cyanide synthesis in *Pseudomonas*, hence the acronym Gac (Haas and Keel 2003). It is now known that Gac regulates the expression of secondary metabolites (including hydrogen cyanide [HCN], phenazines, 2,4-diacetylphloroglucinol [DAPG], pyoluteorin, and the phytohormone indole-3-acetic acid), extracellular enzymes, and several carbon storage compounds as well as an oxidative stress response and other functions that play roles in biological control or plant pathogenicity (Hassan et al. 2010).

Global regulation by Gac in many *Pseudomonas* species was shown to act through the small RNA-binding proteins RsmA and RsmE, members of the RsmA/CsrA family (Reimmann et al. 2005; Lapouge et al. 2008). According to the current model, these small RNA-binding proteins act as negative regulators of gene expression by binding to 5′-GGA-3′ motifs located in the 5′ leading sequence of target mRNAs (Blumer et al. 1999; Heeb and Haas 2001). The binding of these proteins reduces target protein levels either by a reduction in mRNA translation (by blocking the recruitment of the 30S ribosomal subunit), by a reduction in mRNA stability (by targeting messages for degradation), or both (Dubey et al. 2003). Gac also is required for the expression of the small regulatory noncoding RNAs (ncRNAs), *rsmX*, *rsmY*, and *rsmZ* (Lapouge et al. 2008; Humair et al. 2010). These ncRNAs have multiple stemloop structures that interact with RsmA and RsmE, sequestering them, and relieving posttranscriptional repression (Babitzke and Romeo 2007).

The rhizosphere-colonizing bacterium *Pseudomonas chlororaphis* 30-84 is a member of a group of phenazine-producing bacteria with beneficial agronomic applications (Pierson and Pierson 2010). Strain 30-84 is a biological control agent capable of suppressing take-all disease of wheat caused by the fungal pathogen *Gaeumannomyces graminis* var. *tritici* (*Ggt*) (Pierson and Thomashow 1992). Strain 30-84 produces phenazine-1-carboxylic acid (PCA), 2-hydroxy-phenazine-1-carboxylic acid (2OHPCA), and a small amount of 2-hydroxy-phenazine (2OHPZ) (Pierson and Thomashow 1992). Phenazines are responsible for the majority of strain 30-84's ability to inhibit fungal pathogens and have been shown to be important for 30-84 to persist in the rhizosphere (Mazzola et al. 1992) and to form biofilms *in vitro* (Maddula et al. 2006).

Phenazine biosynthesis in 30-84 is controlled by a complex regulatory network involving quorum sensing, Gac and other TCSTs, sigma factor *rpoS*, and other regulatory genes (Pierson and Pierson 2010). Phenazine biosynthesis is regulated directly by the PhzR/PhzI quorum sensing system (Pierson et al. 1994; Wood and Pierson 1996; Wood et al. 1997). PhzR is a LuxR family transcriptional regulator that activates the expression of the phenazine biosynthetic genes in response to the accumulation of *N*-acyl-homoserine lactone (AHL) signals. Mutation in *phzR* abolishes the expression of the phenazine biosynthetic genes and multiple copies of *phzR in trans* significantly increases phenazine production (Pierson et al. 1994). PhzI, a LuxI homolog, is an AHL synthase that produces the AHL signals to which PhzR is most responsive (Wood et al. 1997). In 30-84, mutations in Gac result in loss of phenazine, HCN, exoprotease, lipase, gelatinase, and AHL production as well as losses in the capacity for pathogen inhibition, biofilm formation, and rhizosphere competence (Chancey et al. 1999; Maddula et al. 2006). Other genes with demonstrated roles in phenazine production in *P. chlororaphis* include *pip* (phenazine inducing protein), the *rpeA*/*rpeB* TCST, and *iopA*/*iopB* (inducers of phenazine) (Girard et al. 2006a; van Rij 2006; Wang et al. 2012b). Pip, a homolog of the TetR family of transcriptional regulators (Ramos et al. 2005) was shown to promote phenazine production in 30-84 and *P. chlororaphis* PCL1391 by enhancing *phzI* and *phzR* expression (Girard et al. 2006a; Wang et al. [Bibr b67][Bibr b68]). In both *P. chlororaphis* 30-84 and PCL1391, the expression of *pip* is regulated by the sigma factor *rpoS*. In 30-84, the RpeA/RpeB TCST also regulates *pip* (Wang et al. [Bibr b67][Bibr b68]). In PCL1391, two additional genes, *iopA* and *iopB*, positively contribute to phenazine production through RpoS (van Rij 2006). *Pseudomonas chlororaphis* 30-84 also has a second quorum sensing system, CsaI/CsaR, that controls exoprotease production and cell aggregation, but plays a minor role in phenazine regulation under laboratory conditions (Zhang and Pierson 2001).

GacS/GacA also negatively regulates a spectrum of traits by mechanisms that are as yet largely uncharacterized. For example, inactivation of GacA resulted in the differential expression of 635/6,147 (10%) of the genes in *P. protegens* (*fluorescens*) strain Pf-5 (Hassan et al. 2010). Of these, 288 genes were upregulated in a *gacA* mutant. On solid medium colonies of *gac* mutants often appear hyper-fluorescent and larger than wild type (WT), suggesting that traits related to iron acquisition (e.g., siderophores) or motility may be among the traits negatively regulated by Gac (Chancey et al. 1999). Negative regulation by Gac is particularly interesting given that many *Pseudomonas* species identified for their biological control potential exhibit phenotypic variation resulting from spontaneous mutation in *gacS* or *gacA* (van den Broek et al. 2005). Spontaneous *gac* mutants are especially prominent in fermentation culture, where they often outgrow the WT population resulting in a deficiency of secondary metabolites essential for biological control activity (Duffy and Defago 2000). In comparison, the prevalence of *gac* mutants in natural environments (e.g., in the rhizosphere) suggests some benefit to the Gac^−^ phenotype and thus selection at some level to maintain it. In contrast to liquid culture, in the rhizosphere both the WT (Gac^+^ phenotype) and the Gac mutants (Gac^−^ phenotype) survive better in mixed populations than in uniform populations, demonstrating a benefit to maintaining phenotypic variation (Chancey et al. 2002). More recently, it was shown that the WT and *gac* mutants interact mutualistically in biofilms (Driscoll et al., 2011). The improved survival of both Gac^+^ and Gac^−^ phenotypes in mixed populations suggests that mutants arising de novo or introduced within the inoculum contribute to the survival of 30-84 in the rhizosphere, however, the molecular mechanisms responsible remain unknown.

In this study, the functionality of major components of the Gac/Rsm signal transduction pathway in 30-84 was demonstrated. Transcriptomic analysis using RNA-seq was performed comparing WT and a g*acA* mutant to examine whether the expression of known phenazine regulatory genes including *phzR*/*phzI*, *pip*, *rpoS*, and *rpeA*/*rpeB* were controlled by GacA. We used mutation and complementation analysis of genes within the Gac/Rsm pathway as well as phenazine regulatory genes to determine their hierarchic role in the control of phenazine production. This study expands the current knowledge of the Gac/Rsm regulon, especially its role in phenazine regulation, and based on our results a model describing the regulatory network controlling phenazine gene expression in 30-84 is proposed. The transcriptomic analysis also facilitated the identification of additional genes regulated by GacA that may contribute to the biological control capability of 30-84. Of special interest were genes with increased expression in the *gacA* mutant as these may provide insights into the benefits of the Gac^−^ phenotype observed in mixed populations.

## Experimental Procedures

### Bacterial stains and growth conditions

Bacterial strains and plasmids are listed in Table S1. Oligonucleotides and primers are listed in Table S2. Liquid Luria Broth (LB) medium or AB minimal medium supplemented with 2% casamino acids (AB + 2% CAA) (Difco, Becton Dickinson and Company, Franklin Lakes, NJ) were used for culturing *P. chlororaphis* as described previously (Wang et al. 2012b). The following antibiotics were added to the medium when necessary: ampicillin (Ap) 100 μg/mL, kanamycin (Km) 50 μg/mL, rifampin (Rif) 100 μg/mL, tetracycline (Tc) 50 μg/mL, and gentamicin 30 μg/mL.

### DNA manipulation and sequence analysis

Standard procedures were used for plasmid isolation, cloning, restriction enzyme digestion, and T4 DNA ligation (Sambrook and Russell 2001). Polymerase chain reaction (PCR) was carried out using Invitrogen *Taq* DNA polymerase (Life Technologies, Carlsbad, CA) at 95°C for 5 min, followed by 30 cycles of 95°C for 30 sec, 60°C for 30 sec, and 72°C for 90 sec, and a final elongation step of 70°C for 10 min. DNA sequencing was performed at the Genome Technology Lab within the Texas A&M University Institute for Plant Genomics and Biotechnology. Nucleotide and amino acid homology searches were conducted using the blast programs (BLASTN and BLASTP, respectively) on the National Center for Biotechnology Information (NCBI) website (http://www.ncbi.nlm.nih.gov/BLAST). The *P. chlororaphis* subspecies *aureofaciens* 30-84 genome sequence was recently deposited in GenBank (GenBank Accession #: PRJNA67533). The *P. chlororaphis* 30-84 phenazine biosynthetic operon contains seven conserved biosynthetic genes *phzXYFABCD* (Pierson et al. 1995; Mavrodi et al. 1998), which correspond to *phzABCDEFG* (according to the *P. fluorescens* nomenclature, Mavrodi et al. 1998). Here, we use the *P. chlororaphis* nomenclature to conform to the original literature.

### RNA preparation for RNA-seq and qPCR analyses

Three biological replicates of every strain were started from single colonies located on three separate plates containing AB + 2% CAA and then transferred to 10 mL AB + 2% CAA broth. All cultures were grown at 37°C with shaking (150 rpm) to an approximate OD_600_ = 1.2. Cell cultures collected at OD_600_ = 1.2 were diluted to OD_600_ = 0.3 with AB + 2% CAA broth. RNA extraction was performed as described previously (Wang et al. 2012b) with one exception: contaminating genomic DNA was removed off-column with Turbo DNA-free DNAse (Life Technologies, Carlsbad, CA). Elimination of contaminating DNA was confirmed via qPCR amplification of the *rpoD* gene with SYBR green® dye on an ABI 9400HT PCR machine (Life Technologies, Carlsbad, CA). RNA samples were ethanol-precipitated and resuspended in 0.1% diethylpyrocarbonate (DEPC). Ribosomal RNA (rRNA) was depleted from ∼9 μg of total RNA using the RiboZero rRNA depletion kit (for Gram-negative bacteria, Epicentre Biotechnologies, Madison, WI). Two separate samples were prepared for each treatment using this protocol. At the elution step, the two samples were pooled and concentrated using RiboMinus concentration modules (Life Technologies, Carlsbad, CA). RNA quantification throughout was achieved using a GE NanoVue Plus spectrophotometer (GE Healthcare Bio-Sciences Corp., Piscataway, NJ) and RNA quality was monitored during preparation for library construction and during library construction with an Agilent 2100 Bioanalyzer (Agilent Technologies, Santa Clara, CA) at the Texas A&M GTL.

### Library construction and sequencing

Strand-specific cDNA libraries were constructed using the SOLiD Total RNA-Seq kit according to the manufacturer's instructions with the following modifications: (1) for RNA fragmentation, 300 ng of rRNA-depleted RNA were incubated at 95°C for 5 min. Bioanalyzer traces were used to confirm that the largest proportion of RNA fragments were around 200 bp. (2) Bioanalyzer traces following reverse transcription were used to confirm that hybridization and ligation of RNA adapters and subsequent reverse transcription of the RNA to cDNA was successful. (3) Amplification of cDNA was carried out following reverse transcription, but preceding the size-selection step. Samples were barcoded by replacing the SOLiD 3′ primer with the appropriate barcoding primer as directed in the SOLiD instructions. Size selection of DNA amplicons ranging from 200 to 300 bp was performed using an E-Gel iBase system (SYBR Safe 2% SizeSelect gel) (Life Technologies, Carlsbad, CA). This step was repeated to ensure that adapter–adapter dimers were fully eliminated before sequencing. Paired-end sequencing was carried out by the University of Texas Genomic Sequencing and Analysis Facility on a Life Technologies SOLiD 5500xl sequencing system. Targeted sequencing depth was six-million paired-end reads per sample.

### Read mapping and visualization and transcript quantification

Filtering and alignment of the sequencing data was performed at the UTGSAF using the AB SOLiD BioScope Whole Transcriptome pipeline (v1.3), for whole-transcriptome RNA-seq analysis. Based on gene annotation information, the pipeline produced genome alignment results as a compressed binary version of the Sequence Alignment Map (BAM files). Mapped reads were visualized using BamView in Artemis 13.2.0 (Rutherford et al. 2000).

### RNA-seq data analysis

**Figure 1:**
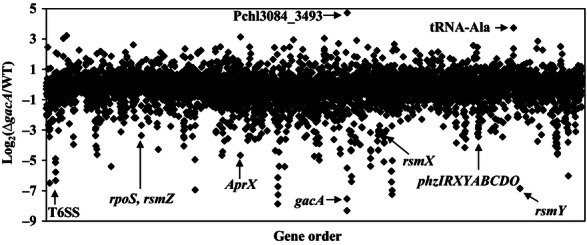
Differential gene expression between wild-type (WT) *Pseudomonas chlororaphis* 30-84 and a *gacA* mutant. Each point represents one of the 5980 annotated genes in the 30-84 genome, with the *x*-axis showing gene order (from the DNA replication origin), and the *y*-axis showing the log_2_ of transcript abundance for each gene in the *gacA* mutant relative to the WT strain. The arrows point to a few of the genes or gene clusters that are differentially expressed. Pchl3084_3493, conserved hypothetical protein; tRNA-Ala, alanine transfer RNA; T6SS, type VI secretion system; *rpoS*, stationary phase sigma factor; *rsmX*, *rsmY*, *rsmZ*: ncRNAs; AprX, metallopeptidase; AprA, metalloprotease.

To determine the transcriptional abundance for each gene, the number of reads that mapped within each annotated sequence was determined. The number of reads per kb of transcript per million mapped reads (RPKM) was used to normalize the raw data (Mortazavi et al. 2008), and mean RPKM values were determined for both the WT and *gacA* mutant samples. The complete dataset from this study has been deposited at the National Center for Biotechnology Information (NCBI) Sequence Read Archive (SRA) with the Accession No. GSE43083. A ratio of the mean RPKM values (*gacA* mutant/WT) was computed for each gene and displayed by gene order from the chromosome origin of replication (Fig. 1). Comparisons were performed using a modified *t*-test and genes with differences in gene expression based on the ratio were considered for further analysis when the *P*-value was less than 0.05 (except as otherwise mentioned) and the expression ratio was ≥2.0 or ≤0.5 (Table S3) (Wang et al. 2011).

### qPCR methods and analysis

qPCR was performed at the Texas A&M GTL using a previously described method (Wang et al. 2012a,b). RNA was reverse transcribed using random primers (Invitrogen) and Superscript III (Invitrogen) at 50°C for 1 h and inactivated at 75°C for 15 min. SYBR Green reactions were performed using the ABI 7900 HT Fast System (Applied Biosystems, Foster City, CA) in 384 well optical reaction plates. Aliquots (1 μL) of cDNA (2 ng/reaction) or water (no-template control) were used as template for qPCR reactions with Fast SYBR Green PCR Master Mix (Applied Biosystems) and primers (500 nmol/L final concentration). Primer pairs gacSRT1-gacSRT2, gacART1-gacART2, rpeBRT1-rpeBRT2, pipRT1-pipRT2, phzYRT1-phzYRT2, phzRRT1-phzRRT2, phzIRT1-phzIRT2, hfqRT1-hfqRT2, rpoDRT1-rpoDRT2, iopBRT1-iopBRT2, 16SRT1-16SRT2, and rpoSRT1-rpoSRT2 were used to detect the expression of *gacS*, *gacA*, *rpeB*, *pip*, *phzY*, *phzR*, *phzI*, *hfq*, *rpoD*, *iopB*, 16S rRNA, and *rpoS* genes, respectively (Table S2). qPCR amplifications were carried out at 50°C for 2 min, 95°C for 10 min, followed by 40 cycles of 95°C for 15 sec and 60°C for 1 min, and a final dissociation curve analysis step from 65 to 95°C. Two technical replicates of each of three biological replicates were used for each experiment. Gene expression levels were analyzed using the relative quantification (ΔΔCt) method (Wang et al. 2011, 2012a,b). The housekeeping sigma factor *rpoD* was used as the reference gene to normalize our samples (ΔCt = Ct_target_ − Ct_rpoD_). A relative quantification (RQ) value was calculated as 2 exp-(ΔΔCt = ΔCt_target_ − ΔCt_reference_) for each gene. A *P*-value was computed using a *t*-test to measure the significance associated with each RQ value. Variations were considered statistically significant when the *P*-value was <0.05. RQ values for the *gacA* mutant were then normalized to those of WT.

### Cloning of *rsmA*, *rsmE*, *rsmX*, *rsmY*, *rsmZ*, and *phzR* genes

The *rsmA* and *rsmE* flanking sequences were used to design primers (rsmA1-rsmA2 and rsmE1-rsmE2) to amplify these genes and their promoter sequences. Following amplification, DNA fragments and vectors (pLAFR3) were digested by *Eco*RI and *Bam*HI and ligated. The resulting plasmids were verified by sequencing and designated as pLAFR3-*rsmA* and pLAFR3-*rsmE*, respectively. Plasmids were introduced into *P. chlororaphis* strains by triparental mating as described previously (Wang et al. 2012b). Transformants were selected on LB plates supplemented with appropriate antibiotics.

Primers pairs rsmX1-rsmX2, rsmY1-rsmY2, rsmZ1-rsmZ2, and phzR1-phzR2 were used to amply *rsmX*, *rsmY*, *rsmZ*, and *phzR* genes. Primers rsmX1, rsmY1, rsmZ1, and phzR1 contain constitutive-hybrid P_*tac*_ promoters (De Boer et al. 1983) that ensure the correct transcription start site. The amplified DNA fragments and vectors (pLAFR3 for ncRNAs and pPROBE-GT2 for phzR) were digested by *Eco*RI and *Bam*HI and ligated resulting in plasmids pLAFR3-P_*tac*_-*rsmX*, pLAFR3-P_*tac*_-*rsmY*, pLAFR3-P_*tac*_-*rsmZ*, and pGT2*-*P_*tac*_*-phzR*. The genotypes were confirmed by both enzymatic digestion and sequencing.

### Construction of an *rsmE* mutant

A 30-84 genomic library was introduced into strain 30-84ZN (*phzB::lacZ*) via triparental mating. Exconjugants were screened for alterations in phenazine gene expression as indicated by differences in β-galactosidase activity on medium containing X-gal. Cosmid pLSP298 caused a visible reduction in *phzB* expression and was chosen for further analysis. Cosmid pLSP298 was mutated using EZ::TN <KAN-2> (Epicentre Biotechnologies, Madison, WI) and cosmids containing insertions were introduced into strain 30-84ZN via triparental mating and compared with strain 30-84ZN (pLSP298) for *phzB::lacZ* expression. One EZ::TN insertion (pLSP298EZ::TN5-11-2) that no longer reduced *phzB* expression was cloned as a *Bam*HI fragment containing the EZ::TN Km^R^ gene into pIC20H (Marsch et al. 1984). The regions adjacent to the cloned region of the EZ::TN were sequenced using the EZ::TN supplied primer and shown to be *rsmE*. Cosmid pLSP298EZ::TN5-11-2 was introduced into strain 30-84ZW, a Δ*gacA* β-galactosidase reporter (*phzB::lacZ, gacA*^*−*^) via triparental mating. As a result, the *rsmE*::EZ::TN insertion was introduced into the genome of 30-84ZW via marker exchange to create a Δ*gacA* β-galactosidase reporter with a mutation in *rsmE*, designated 30-84ZWE (*phzB::lacZ*, *gacA*^*−*^, *rsmE*^*−*^).

### Quantification of *rsmZ* promoter activity

A 630 bp *Sal*I fragment immediately upstream of *rsmZ* that contained the *rsmZ* promoter (P_*rsmZ*_) was cloned into the *Sal*I site within pIC20H. A 681 bp *Eco*R1-*Hin*dIII fragment from the resulting plasmid was cloned into pPROBE-KT2 upstream of the promoter-less GFP gene to create pKT2-P_*rsmZ*_-*gfp*. A *Hin*dIII fragment containing a promoter-less *lacZ* gene was cloned from plasmid pKOK6.1 and inserted into the unique *Hin*dIII site between P_*rsmZ*_ and *gfp* in pKT2-P_*rsmZ*_-*gfp* resulting in pKT2-P_*rsmZ*_-*lacZ*-*gfp*. Plasmid pKT2-P_*rsmZ*_-*lacZ*-*gfp* was introduced into the 30-84Ice strain and *gac* mutants via triparental mating. GFP expression for strains containing pP_*rsmZ*_-*lacZ*-*gfp* was determined visually and quantified by β-galactosidase activity (Miller 1972).

### Quantification of phenazine production

*Pseudomonas chlororaphis* strains were grown with aeration at 28°C in LB for 24 h. Phenazines were extracted and quantified by UV-visible light spectroscopy as described previously (Wang et al. [Bibr b67][Bibr b68]). Briefly, triplicate 10 mL cultures grown overnight at 28°C were centrifuged (5000*g*), and the supernatants were acidified to ca. pH 2 with concentrated HCl. Phenazines were extracted with an equal volume of benzene for 6 h. Following separation and evaporation of the benzene phase under a stream of air, phenazines were resuspended in 0.5 mL of 0.1 N NaOH, and serial dilutions were quantified via absorbance at 367 nm. The absorbance for each sample was normalized to the total absorbance of the 10 mL culture. Strain 30-84ZN, used as a negative control, carries a mutation in the *phzB* gene and is deficient in phenazine production.

### AHL extraction and biological assays

Total AHL extractions were prepared from cell-free supernatants of strain 30-84, Δ*gacS*, or Δ*gacA* with plasmid pLAFR3 containing either no insert (NI) or *rsmZ*. The cell-free supernatants were made from overnight cultures grown in LB medium (OD_600_ ∼1.8) as described previously (Pierson et al. 1998). Briefly, 5 mL cultures were grown overnight at 28°C with shaking in LB broth to an OD_600_ of 1.8. The cultures were centrifuged and supernatants were mixed with an equal volume of acidified ethyl acetate. The ethyl acetate phase was evaporated and the dried extracts containing AHLs were suspended in a volume of LB equal to the original culture, which was subsequently filter-sterilized (0.45 μm). AHL production was quantified by inoculating the extracted AHLs with the AHL-specific reporter *P. chlororaphis* strain 30-84I/Z (*phzI*^***−***^, *phzB::lacZ*). Strain 30-84I/Z is deficient in AHL production, but responds to exogenously added AHL's by producing β-galactosidase. β-galactosidase activity was determined subsequently on cultures grown shaking at 28°C after 24 h (Wood and Pierson 1996). The assays were repeated at least three times.

### Phenotypic analyses and fungal inhibition assays

The production of siderophore, exoprotease, gelatinase, amylase, lipase, and HCN was qualitatively measured (Chancey et al. 1999, 2002). Strains were spotted onto appropriate media: KMB plates + FeCl_3_·6H_2_O (final concentration 1 mmol/L) for fluorescence, skim milk plates for exoprotease, gelatin plates for gelatinase, starch plates for amylase, and tributyrin plates for lipase, respectively. For HCN production, a 1-inch piece of cyantesmo cyanide indicator paper (Macherey-Nagel, Germany) was attached to the inside lid of sealed plates. Cell growth and phenotypes were measured after 24 and 48 h. For pathogen inhibition, overnight cultures of 30-84 and derivatives were spotted onto triplicate LB + 0.5% potato dextrose agar plates. After 2 days of growth at 28°C, a 5 mm plug of *G. graminis* var*. tritici* was placed in the center of the plates. After 4 days, zones of inhibition, the distance between the edge of the bacterial colony and the fungal mycelium, were measured (Whistler and Pierson 2006). The assays were repeated twice.

### Bacterial swimming motility assays

For *P. chlororaphis* WT, *gac* mutants, and 30-84Z strain, bacterial cell suspensions were grown overnight in LB broth. Five microliter of the bacterial suspension was plated onto the center of motility agar plates (10 g tryptone, 5 g NaCl, 2.5 g agar per liter distilled water) as previously described (Wang 2011). Diameters were determined following incubation at 28°C for 48 h. The experiments were repeated at least three times.

## Results and Discussion

### Transcriptomic analyses of phenazine biosynthetic and regulatory genes

The RNA-seq analysis identified 771 genes that were differentially expressed in the *gacA* mutant compared with the WT (Fig. 1; Table S3). Among these genes, the transcripts of 551 genes were underrepresented and 220 genes were overrepresented in the *gacA* mutant relative to WT. Genes encoding components of the Gac signal transduction pathway were among those differentially regulated (Fig. 1).

#### Transcript abundance patterns in *P. chlororaphis* 30-84 WT

**Figure 2:**
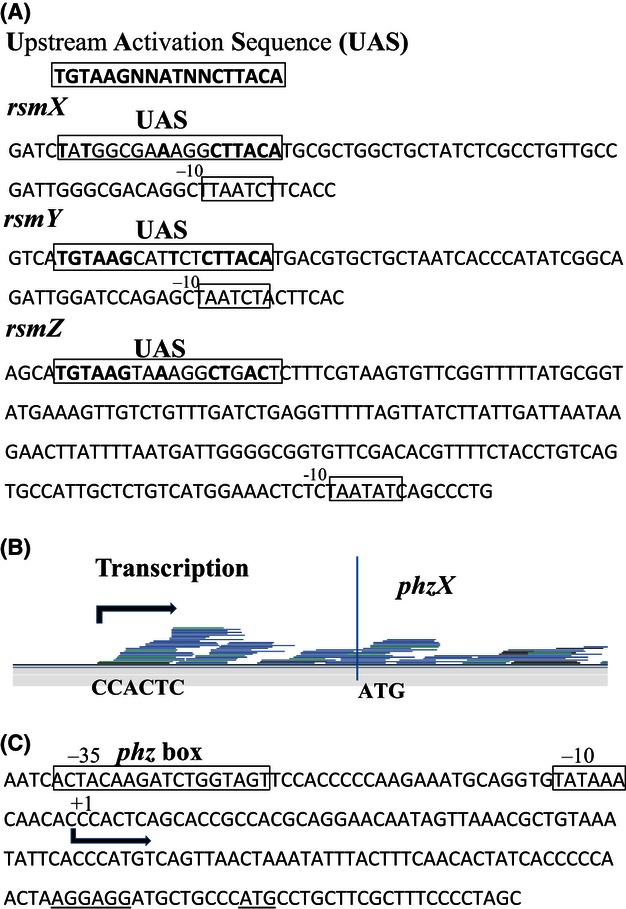
Promoter regions of ncRNA and phenazine biosynthetic genes. (A) Alignment of the *rsmX*, *rsmY*, and *rsmZ* promoter regions of *Pseudomonas chlororaphis* 30-84. The consensus Upstream Activation Sequence recognized by GacA (UAS) (Humair et al., 2010) is shown. The putative UAS for each promoter region is boxed and conserved nucleotides are in bold. The −10 promoter elements are also indicated. (B) RNA-seq profile showing sequence reads across the *phz* promoter region. RNA-seq results were visualized using the Artemis (http://www.sanger.ac.uk/resources/software/artemis/) genome browser. Transcription and translation start sites are shown below the profile. (C) Nucleotide sequence of the *phz* promoter. Boxed nucleotides indicate sequences involved in transcription initiation (PhzR Box, RNA polymerase binding site), whereas sequences involved in translation initiation (ribosome or RsmE binding sites) regulatory elements are underlined. Arrow indicates the direction of transcription from the start site.

To better understand the role of the Gac system on phenazine regulation, transcript abundance of the phenazine biosynthetic and regulatory genes in the WT were measured. The mean RPKM determined by RNA-seq for *gacA* and *gacS* in the *P. chlororaphis* 30-84 WT were 266.9 and 29.2 (Table 1), respectively, demonstrating that in the WT, *gacA* expression is about ninefold higher than *gacS* under these experimental conditions (e.g., grown in AB + 2% CAA to OD_600_ = 1.2). RPKM values for *rsmA* and *rsmE* in WT were similar to each other (151.8 and 292.7, respectively), and to *gacA*, whereas values for the three ncRNAs *rsmX*, *rsmY*, and *rsmZ* were significantly higher. In *P. protegens* Pf-5, GacA transcriptionally activates *rsmX*, *rsmY*, and *rsmZ* by binding to conserved motifs in their promoter regions (Humair et al. 2010). Analysis of *rsmX*, *rsmY*, and *rsmZ* in *P. chlororaphis* 30-84 revealed the conserved palindromic consensus sequence typical of GacA-controlled ncRNA genes and a conserved −10 hexamer (TAATCT) promoter sequence identical to that found in *P. protegens* Pf-5 (Humair et al. 2010) (Fig. 2A). Analogous to *P. protegens* Pf-5, the *rsmX* and *rsmY* promoter sequences are similar in length, but the *rsmZ* promoter sequence is longer (Fig. 2A). Consistent with *rsmY* having a promoter with the highest similarity to the conserved GacA binding site, *rsmY* was expressed at the highest level (RPKM = 38112.4), approximately 22-fold higher than *rsmX* (RPKM = 1486.5) or *rsmZ* (RPKM = 1679.6) in the WT (Table 1).

**Table 1:** Mean transcript abundance and ratio of abundances (*ΔgacA*/WT) of the phenazine biosynthetic and regulatory genes in the *gacA* mutant compared with the WT strain

Gene ID	Gene	Protein description	Mean RPKM WT	Mean RPKM *ΔgacA*	*ΔgacA*/WT	*P*-value
*gacS*/*gacA*						
Pchl3084_3491	*gacA*	Response regulator	266.9	1.43	0.01	0.00
Pchl3084_4333	*gacS*	Sensor protein	29.17	21.15	0.73	0.05
*phz* genes						
Pchl3084_4951	*phzX*	Phenazine biosynthesis protein	40.50	4.52	0.11	0.03
Pchl3084_4952	*phzY*	Phenazine biosynthesis protein	37.87	5.28	0.14	0.03
Pchl3084_4953	*phzF*	Phenazine biosynthesis protein	20.00	2.63	0.13	0.03
Pchl3084_4954	*phzA*	Phenazine biosynthesis protein	8.52	0.78	0.09	0.15
Pchl3084_4955	*phzB*	Phenazine biosynthesis protein	13.47	2.08	0.15	0.04
Pchl3084_4956	*phzC*	Phenazine biosynthesis protein	8.24	1.90	0.23	0.00
Pchl3084_4957	*phzD*	Phenazine biosynthesis protein	7.63	1.40	0.18	0.10
Pchl3084_4958	*phzO*	Phenazine biosynthesis protein	44.23	21.70	0.49	0.06
Regulatory genes						
Pchl3084_4949	*phzI*	Autoinducer synthase	5.24	1.50	0.29	0.01
Pchl3084_4950	*phzR*	Transcriptional activator	125.1	67.12	0.54	0.02
Pchl3084_1189	*rpoS*	Sigma factor	1045.7	101.8	0.10	0.03
Pchl3084_5155	*pip*	Phenazine-inducing protein	123.4	105.4	0.85	0.04
Pchl3084_1659	*iopA*	Inducer of phenazine A	49.10	8.35	0.17	0.01
Pchl3084_1660	*iopB*	Inducer of phenazine B	20.71	2.51	0.12	0.00
Pchl3084_3224	*rpeA*	Sensor histidine kinase	46.78	49.28	1.05	0.67
Pchl3084_3225	*rpeB*	Response regulator	45.37	55.20	1.22	0.07
Pchl3084_2449	*csaI*	Autoinducer synthase	8.15	8.72	1.07	0.65
Pchl3084_2451	*csaR*	Transcriptional activator	0.54	0.38	0.71	0.50
Pchl3084_0554	*hfq*	RNA chaperone	1642.19	1408.2	0.84	0.28
Pchl3084_4387	*rsmA*	Translational regulator	151.82	307.24	2.02	0.03
Pchl3084_2024	*rsmE*	Translational regulator	292.7	204.83	0.70	0.06
Pchl3084_3970	*rsmX*	ncRNA	1486.51	201.13	0.14	0.02
Pchl3084_5419	*rsmY*	ncRNA	38112.4	329.2	0.01	0.02
Pchl3084_1190	*rsmZ*	ncRNA	1679.64	261.26	0.16	0.05

We also looked at the relative transcript abundance of other regulatory genes associated with phenazine production or quorum sensing (Table 1). In the WT, *hfq* and *rpoS* were expressed at very high levels with RPKM values of 1642.2 and 1045.7 (the average RPKM value is 119.5), respectively. These two regulators were of interest because the RNA chaperone, Hfq, has been shown previously to control *luxR*-type regulators (Wilf et al. 2012) and the sigma factor, RpoS, to control *phzR*/*phzI* through *pip* (Girard et al. 2006a; Wang et al. 2012b). RPKM values for the two essential phenazine regulators *pip* and *phzR* were 123.4 and 125.1, around 10-fold lower than those of *hfq* and *rpoS*. The RPKM value for the other quorum sensing regulator *csaR*, which plays a minor role in phenazine regulation under laboratory conditions, was only 0.54 suggesting it was barely expressed under the assay conditions. The RPKM values for both quorum sensing signal synthases, *phzI* and *csaI*, were 5.2 and 8.2, respectively.

Similar to other phenazine-producing bacteria, the core phenazine, PCA, is encoded by a conserved set of biosynthetic genes (Mavrodi et al. 2006; Mentel et al. 2009); in 30-84 these are *phzXYFABCD* (Pierson et al. 1995). According to the RNA-seq analysis, the transcription start site of the *phzX* was predicted 115 nt upstream of the translation start site (ATG) (Fig. 2B). This was consistent with the expected structure of a quorum sensing-regulated promoter containing a −10 hexamer and a Lux-box located at the −35 site predicted for PhzR binding (Qin et al. 2007) (Fig. 2C). The transcript abundance of the *phz* genes showed a staircase-like pattern from *phzX* to *phzD* (Table 1), suggesting decreased transcription of genes with position along the *phz* operon. In many phenazine-producing *Pseudomonas* species, PCA is then modified into other derivatives by one or more accessory genes encoding modifying enzymes (Mavrodi et al. 2006). In 30-84, PCA is modified into the 2-hydroxy forms, 2OHPCA and 2OHPZ, by an enzyme encoded by *phzO*, adjacent to the phenazine biosynthetic operon (Maddula et al. 2008). It was shown previously that the expression of *phzO* was driven by its own promoter (Maddula et al. 2008) and this was supported by the RNA-seq analysis (data not shown). The expression of *phzO* was similar to that of *phzX* (RPKM values of 40.5 and 44.2, respectively, Table 1).

#### Phenazine biosynthetic and regulatory gene transcripts are in lower abundance in the *gacA* mutant relative to WT

In order to identify genes regulated by GacA in 30-84, mean RPKM values for the *gac*A mutant were compared with WT values. As expected, the RPKM value for *gacA* was very low in the *gacA* mutant compared with the WT (1.43 vs. 266.9, respectively) demonstrating that the *gac*A message was successfully disrupted, whereas expression of *gacS* was unchanged (Table 1). Mutation in *gacA* also resulted in lower abundance of the three ncRNAs; the greatest fold change was for *rsmY* (∼115-fold decrease). The RPKM values for *rsmX*, *rsmY*, and *rsmZ* were decreased to 201.1, 329.2, and 261.3, respectively (Table 1). These data indicate that GacA positively regulates the expression of *rsmX*, *rsmY*, and *rsmZ* in *P. chlororaphis* 30-84. Interestingly, the transcript abundance of *rsmA* was 2.2-fold higher in the *gacA* mutant compared with WT 30-84, whereas that of the closely related *rsmE* was relatively unchanged (Table 1).

**Figure 3:**
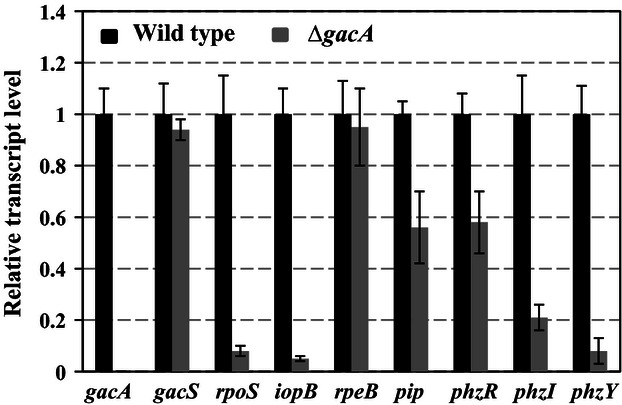
Verification of RNA-seq data by quantitative reverse transcription-polymerase chase reaction (qPCR). The relative fold change of *gacA*, *gacS*, *rpoS*, *iopB*, *rpeB*, *pip*, *phzI*, *phzR*, and *phzB* genes in *gacA* mutant compared towith the WT strain. The gene *rpoD* was used as the reference. Cells were grown in AB minimal medium + 2% casamino acid for 18 h with shaking to an OD_600_ of 1.2. Data points represent means of three replicates ± standard deviations. These experiments were repeated at least three times and similar results were obtained.

Consistent with the deficiency in phenazine production observed in *gac* mutants, mutation in *gacA* resulted in 2- to 11-fold decreases in the transcript abundance of the phenazine biosynthetic genes (Table 1). Transcripts of six regulatory genes involved in phenazine production, *iopA*, *iopB*, *rpoS*, *pip*, *phzR*, and *phzI*, were underrepresented by one- to ninefold in the *gacA* mutant as compared with WT, whereas other regulatory genes such as *hfq*, *csaI*/*R*, and *rpeA*/*rpeB* were not appreciably altered (Table 1). The expression of these phenazine regulatory and biosynthetic genes was verified by qPCR (Fig. 3). The results suggest that GacA controls the expression of the phenazine biosynthesis genes in 30-84 partly by activating the expression of *phzR*, *phzI*, *pip*, *rpoS*, and *iopA*/*iopB*.

### Genetic analyses of the phenazine biosynthetic and regulatory genes in 30-84

#### Expression of *phzR*/*phzI* does not rescue phenazine production in a *gacA* mutant

**Figure 4:**
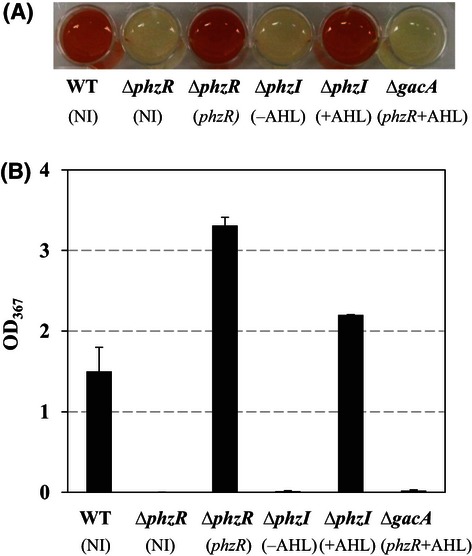
Phenazine production by WT and mutants with additional copies of *phzR* and supplemental AHLs. (A) Bacterial strains were grown in 2 mL LB medium in a 24-well plate at 28°C without shaking. The photo was taken 48 h after inoculation. (B) Bacterial strains were grown in LB medium for 24 h at 28°C with shaking at 200 rpm. Treatments included strains with the plasmid pGT-2 containing either no insert (NI) or the plasmid containing a P_*tac*_-*phzR* insertion, AHLs were added to the growth medium as indicated. Phenazine was extracted and quantified at OD_367_. AHLs were extracted as in ethyl acetate as described previously and total AHL quantified by ability to rescue phenazine production in the *phzI* mutant 30-84I. Data points represent means of three replicates ± standard deviations.

It was shown previously that the expression of *phzI* and AHL production are significantly reduced in 30-84 *gacS* and *gacA* mutants (Chancey et al. 1999). RNA-seq analysis indicated that both quorum sensing genes *phzI* and *phzR* were significantly decreased in the *gacA* mutant. One hypothesis is that this deficiency in quorum sensing is the reason for the lack of phenazine production by *gacA* mutants. However, if Gac operates by controlling phenazine production posttranscriptionally, constitutive expression of quorum sensing genes should not complement a *gac* mutant. To test this, the *phzR* gene was cloned into a medium copy vector driven by the P_*tac*_ promoter, and the plasmid with or without the *phzR* insertion was introduced into either WT, 30-84R (*phzR*^*−*^), or the *gacA* mutant. As expected, complementation of the *phzR* mutant by the pGT2-P_*tac*_-*phzR* plasmid or the *phzI* mutant 30-84I by the addition of AHLs fully rescued phenazine production (Fig. 4A and B). In contrast, constitutive expression of *phzR* with the addition of AHLs to the growth medium did not rescue phenazine production in the *gacA* mutant (Fig. 4A and B). These results demonstrate that the underexpression of the PhzR/PhzI quorum sensing system in Gac mutants is not solely responsible for the phenazine deficiency observed.

#### RsmE, but not RsmA, regulates phenazine and AHL production

**Figure 5:**
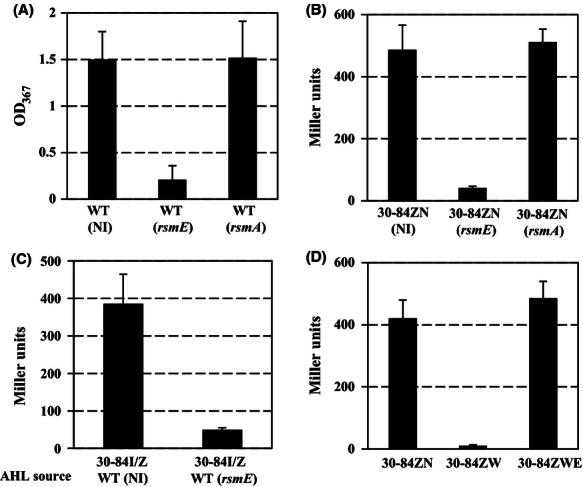
Role of RsmE in AHL and phenazine production. (A) Phenazine production by 30-84 with plasmid pLAFR3 containing either no insert (NI), *rsmA*, or *rsmE*. Bacterial strains were grown in LB medium for 24 h at 28°C with shaking. Phenazine was extracted as described previously and total phenazine was quantified at OD_367_. Data points represent means of three replicates ± standard deviations. (B) Repression of *phzB* expression in 30-84ZN by introduction of extra copies of *rsmE* but not by introduction of extra copies of *rsmA*. Treatments included 30-84ZN with plasmid pLAFR3 containing either NI, *rsmE*, or *rsmA*. 30-84ZN is a translational *phzB::lacZ* fusion. The β-galactosidase activities (Miller Units) were determined in triplicate (mean ± SE). (C) Suppression of AHL production by *rsmE* in trans. AHL accumulation was quantified from overnight cultures of WT with pLAFR3 containing either NI or *rsmE* using the AHL-specific reporter 30-84I/Z (*phzI*^*−*^, *phzB::lacZ*). The relative amount of AHL was determined by β-galactosidase assays. (D) Effect of inactivation of *rsmE* on *phzB* expression. Treatments included the *phzB::lacZ* reporter (30-84ZN), a *phzB::lacZ*/GacA^−^ derivative (30-84ZW), and a *phzB::lacZ*/GacA^−^/RsmE^−^ derivative (30-84ZWE). Bacterial cultures were grown in LB and *phzB* expression was measured as β-galactosidase activity after 24 h. Data are the means of two replicates from one representative experiment.

*Pseudomonas chlororaphis* 30-84 has both of the RNA-binding proteins RsmA and RsmE (this study) and they share 72% and 87% sequence identity and similarity, respectively. Amino acid identity of 30-84 RsmA and RsmE to their respective homologs in other *Pseudomonas* species was high (e.g., 100% and 93% identical to *P. protegens* Pf-5, *P. fluorescens* Pf01, *P. syringae* DC3000, *P. aeruginosa* PAO1; 94% and 91% identical *Escherichia coli* O157). The current model predicts that overexpression of RsmA or RsmE in the WT should provide phenotypes that are similar to *gac* mutants (Heeb and Haas 2001). To determine the roles of RsmA and RsmE in the regulation of phenazine production, multiple copies of *rsmA* and *rsmE* were introduced separately into the WT strain. Phenazine production decreased 7.1-fold in the WT strain harboring pLAFR3-*rsmE* (compared to WT with pLAFR3-NI) whereas overexpression of *rsmA* did not alter phenazine production in the WT strain (Fig. 5A). To confirm that *rsmA* was overexpressed in the WT, qPCR was performed and results showed that multiple copy of *rsmA* leads to 12.8 ± 0.6-fold transcript increase in the WT strain. To correlate phenazine production with phenazine gene expression, *phzB::lacZ* expression was measured in 30-84ZN harboring these plasmids. Consistent with phenazine production, multiple copies of *rsmE* reduced 30-84ZN β-galactosidase activity from 487.0 ± 78.6 to 48.1 ± 5.2 in 30-84ZN with pLAFR3-NI compared with pLAFR3-*rsmE*, respectively; the -galactosidase activity produced by strain 30-84ZN harboring pLAFR3-*rsmA* was similar to the 30-84ZN (pLAFR3-NI) control (511.0 ± 42.1 vs. 487.0 ± 78.6, respectively) (Fig. 5B). The result that RsmE, but not RsmA is involved in the regulation of phenazines is consistent with the observation in *Pseudomonas* sp. M18 that RsmA negatively controls pyoluteorin production but not phenazine production (Zhang et al. 2005). As pyoluteorin genes were not detected in *P. chlororaphis* 30-84, the target(s) of RsmA remains to be discovered.

To determine whether RsmE also inhibited AHL production, the effect of overexpression of *rsmE* on AHL signal production was measured. Total AHL signal was extracted from 24 h bacterial cultures of WT (pLAFR3-NI) and WT (pLAFR3-*rsmE*). The relative amount of AHL signal was quantified by activation of β-galactosidase activity in strain 30-84IZ (Fig. 5C). β-galactosidase activity of strain 30-84IZ was approximately eightfold lower when the reporter strain was grown in the presence of AHLs prepared from overnight cultures of WT (pLAFR3-*rsmE*) compared with AHLs prepared from WT (pLAFR3-NI). These results indicate that RsmE overexpression also resulted in a significant decrease in AHL production.

To determine whether RsmE functions downstream of GacA in the regulation of phenazine gene expression, cosmid pLSP298EZ:TN5-11-2 containing a transposon insertion that inactivated *rsmE* was introduced into the genome of the *gacA* mutant 30-84ZW via marker exchange mutagenesis. Comparison of *phzB::lacZ* expression in strains 30-84ZN, 30-84ZW, and 30-84ZWE indicated that loss of *rsmE* restored β-galactosidase activity to the *gacA* mutant (Fig. 5D).

#### Constitutive expression of *rsmZ* rescues phenazine and AHL production in *gac* mutants

**Figure 6:**
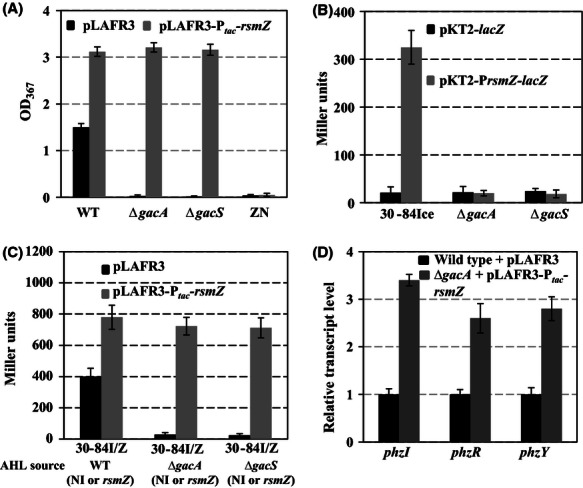
Impact of *rsmZ* on phenazine and AHL production. (A) Phenazine production by different derivatives in vitro. Treatments included strains with plasmid pLAFR3 containing either no insert (NI) or P_*tac*_-*rsmZ*. Growth conditions were as in (Fig. 5A). Phenazine was extracted in benzene and the amount of phenazine was measured at OD_367_. Data points represent means of three replicates ± standard deviations. Similar results were obtained in at least three independent experiments. (B) Promoter activity of the *rsmZ* gene in WT, *gacS*, and *gacA* was determined by β-galactosidase activities. Treatments included strains with plasmid pKT-2 containing either a promoter-less *lacZ* insertion or the *rsmZ* promoter fused to the promoter-less *lacZ* gene (P_*rsmZ*_*-lacZ*). Insertions were made in front of the promoter-less *gfp* gene, already present on the plasmid. Bacterial strains were grown in LB medium for 24 h at 28°C with shaking to an OD_600_ of ∼1.8. Each value is the average from two different cultures ± standard deviations with two independent experiments. The experiment was repeated at least twice and similar results were obtained. (C) Effect of *rsmZ* on AHL production. Total AHL extractions were prepared from cell-free supernatants of WT, Δ*gacS*, or Δ*gacA* with plasmid pLAFR3 containing either NI or P_tac_-*rsmZ*. Cell-free supernatants were made from overnight cultures grown in LB medium (OD_600_ ∼1.8). The relative amount of AHL in each extract was determined by β-galactosidase assays using the AHL-specific reporter 30-84I/Z (*phzI*^*−*^, *phzB::lacZ*). Bars represent β-galactosidase activity measured in Miller Units of 30-84I/Z grown with the AHL extracts indicated. Each value is the average from three different cultures ± standard deviations. (D) Relative expression of *phzI*, *phzR*, and *phzY* genes in the *gacA* mutant (harboring the pLAFR3-P_*tac*_-*rsmZ*) compared with WT (harboring pLAFR3 empty vector) as determined by qPCR. *rpoD* was used as the reference gene. Cells were grown in AB minimal medium + 2% casamino acid for 18 h with shaking to an OD_600_ of 1.2. Data points represent means of three replicates ± standard deviations. These experiments were repeated at least three times and similar results were obtained.

We further examined whether constitutive expression of ncRNAs would remove the need for a functional Gac system for phenazine production in strain 30-84. The *rsmX*, *rsmY*, and *rsmZ* genes were cloned under the control of a constitutive P_*tac*_ promoter such that the +1 site of the promoter was at the beginning of these genes. Although strains *ΔgacS* (pLAFR3-NI) and *ΔgacA* (pLAFR3-NI) failed to synthesize measurable amounts of phenazines, strain *ΔgacS* (pLAFR3-P_*tac*_-*rsmZ*) and *ΔgacA* (pLAFR3-P_*tac*_-*rsmZ*) produced approximately the same amount of phenazines as WT (pLAFR3-P_*tac*_-*rsmZ*) (Fig. 6A). In contrast, constitutive expression of *rsmX* did not rescue phenazine production (data not shown). The expression *rsmX* was confirmed using qPCR, the *gacA* mutant containing *rsmX* plasmid showed 2.4-fold increase of *rsmX* expression than the WT strain. Constitutive expression of *rsmY* by the P_*tac*_ promoter appeared to be lethal in *gac* mutants, as no transformants were recovered with electroporation or triparental mating (data not shown).

We further explored the regulatory role of GacA on ncRNA expression by characterizing the effect of mutations in *gacA* on *rsmZ* promoter activity using the reporter plasmid pKT2-P_*rsmZ*_-*lacZ-gfp* (e.g., by measuring fluorescence or β-galactosidase activity). The reporter plasmid was introduced into a *gacA* and a *gacS* mutant as well as 30-84Ice (*phzB::inaZ*), which has a functional *gacA*, but does not produce phenazines (thus no interference with GFP fluorescence). Strain 30-84Ice (pKT2-P_*rsmZ*_-*lacZ-gfp*) was brightly fluorescent whereas the Gac mutants with the reporter plasmid were not (data not shown). Introduction of the plasmid into the *gacA* or *gacS* mutant resulted in an 11-fold reduction in expression of β-galactosidase relative to strain 30-84Ice (pKT2-P_*rsmZ*_-*lacZ-gfp*) (Fig. 6B). These results confirm that the Gac system positively regulates the expression of *rsmZ*.

To determine whether constitutive expression of *rsmZ* also regulated AHL signal production, the effect of constitutive expression of *rsmZ* on AHL signal production was measured. Total AHL signal was extracted from 24 h bacterial cultures and the relative amount of AHL signal present was quantified by activation of β-galactosidase activity in strain 30-84IZ (Fig. 6C). The β-galactosidase activity of the 30-84IZ reporter was significantly higher when grown with AHLs prepared from strains *ΔgacS* (pLAFR3-P_*tac*_-*rsmZ*) and *ΔgacA* (pLAFR3-P_*tac*_-*rsmZ*) compared with AHLs prepared from *ΔgacS* (pLAFR3-NI) and *ΔgacA* (pLAFR3-NI); β-galactosidase activities of the 30-84IZ reporter were 712.0 ± 77.1 and 723.1 ± 56.2 as compared with 28.9 ± 12.2 and 24.1 ± 10.2, respectively. This increase (27-fold) in *phzB* expression with constitutive *rsmZ* expression supports the hypothesis that the presence of the *rsmZ* ncRNA bypasses the requirement for a functional Gac system for both phenazine and AHL production. qPCR analyses confirmed that the transcript abundance of *phzI*, *phzR*, and *phzY* in the *gacA* mutant was two- to fourfold higher in the presence of *rsmZ* (Fig. 6D). Collectively, these results indicate that Gac regulates phenazine production and quorum sensing by activating the expression of *rsmZ*.

Constitutive expression of *rsmZ* also qualitatively restored HCN, exoprotease, gelatinase, and lipase production and reduced fluorescence to WT levels in strain 30-84W (data not shown). Consistent with restoration of these capabilities, constitutive expression of *rsmZ* also reinstated 30-84's ability to inhibit the mycelial growth of the fungal pathogen *Ggt*. Zones of *Ggt* mycelial inhibition by WT, *ΔgacA*, *ΔgacA* (pLAFR3-NI), and *ΔgacA* (pLAFR3-*rsmZ*) were 8.4 ± 0.9, 0.0 ± 0.3, 0.2 ± 0.4, and 9.4 ± 0.8 mm, respectively.

### Model of Gac/Rsm regulation of phenazines in 30-84

**Figure 7:**
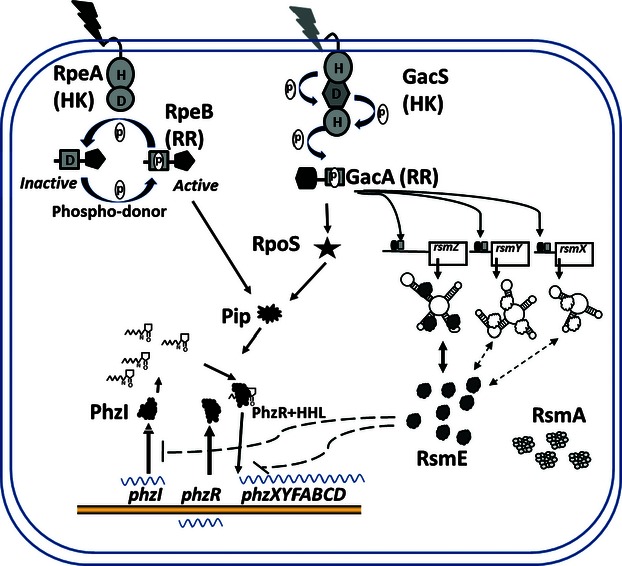
Proposed model for the regulation of phenazine biosynthesis by the Gac-Rsm system in *Pseudomonas chlororaphis* strain 30-84. The model shows the presumed position of the GacS/GacA system in relation to other known regulators of phenazine production. GacS is a transmembrane protein according to in silico predictions based on amino acid sequences. Amino acids H (histidine) and D (aspartate) involved in phosphorylation are indicated. The shaded area of GacA indicates the DNA-binding domain. Solid arrows and blunt lines point to genes (or processes) that are positively or negatively affected, respectively. Dashed arrows or lines indicate unknown or as yet uncharacterized regulatory pathways. GacA positively controls the expression of *rsmX*, *rsmY*, and *rsmZ*, which in turn activates phenazine production by titrating the translation suppressor RsmE. Although the transcript abundance of *rsmA* was 2.2-fold higher in the *gacA* mutant compared with WT 30-84, RsmA does not appear to play a role in the regulation of phenazines under the experimental conditions tested here. A functional GacA is also required for increased expression of other phenazine regulatory genes, including *rpoS*, *pip*, and *phzR*/*phzI*. HK, histidine kinase; RR, response regulator; P, phosphoryl group.

In this study, the major components of the Gac/Rsm signal transduction pathway in *P. chlororaphis* 30-84 were identified and characterized (Fig. 7). For example, we discovered that the genome of 30-84 contains genes encoding both RsmE and RsmA. Only overexpression of *rsmE* in the WT reduced the gene expression and production of phenazine. Similarly only deletion of *rsmE* in a *gacA* mutant restored both, demonstrating that *rsmE* deletion bypassed the need for a functional GacA to produce phenazines. The genome of 30-84 contains the gene sequences for the three small ncRNAs, *rsmX*, *rsmY*, and *rsmZ*. A functional Gac system is required for *rsmZ* expression and that expression of *rsmZ* from a constitutive promoter restored phenazine, HCN, exoprotease, lipase, and gelatinase production and reduced fluorescence to WT levels in a *gacA* mutant. In contrast, constitutive expression of *rsmX* did not restore any of the phenotypes tested, and constitutive expression of nor *rsmY* could not be achieved. The importance of the *rsmZ* in Gac regulation of products essential for biological control is interesting given that *rsmZ* has the longest promoter region, suggesting the potential for multiple protein interactions in its regulation. The observation that *rsmZ* plays a different role than the other two ncRNAs in Gac-mediated regulation has not been reported previously. However, the observation that transcript abundances of both *rsmX* and particularly *rsmY* are significantly lower in a GacA mutant is consistent with the hypothesis that they are involved in Gac-mediated regulation of as yet unidentified targets. Consistent with Gac/Rsm exerting posttranscriptional control of phenazine production, previous work showed that the introduction of a cosmid containing the entire phenazine biosynthetic cluster and *phzR* and *phzI* in trans on a low copy plasmid failed to complement a *gacA* mutant for phenazine production (Chancey et al. 1999). In this study, we bypassed transcriptional regulation of quorum sensing by providing PhzR (from a constitutive promoter) and purified AHL signal, confirming the prediction that Gac/Rsm controls phenazine gene expression posttranscriptionally.

The transcriptomic data demonstrated that GacA controls the expression of the phenazine biosynthetic genes (*phzXYFABCD* and *phzO*), the quorum sensing genes *phzR and phzI*, and also several additional regulatory genes involved in phenazine gene expression including *pip*, *rpoS*, and *iopA*/*iopB*. In *P. chlororaphis* stains 30-84 and PCL1391, Pip promotes phenazine production by enhancing *phzI* and *phzR* expression (Girard et al. 2006a; Wang et al. [Bibr b67][Bibr b68]). In both strains, the expression of *pip* is regulated by the sigma factor *rpoS*, which in turn is regulated by GacA. In 30-84 *pip* also is regulated by the RpeB/RpeA TCST system (Wang et al. [Bibr b67][Bibr b68]). Complementation assays showed that constitutive expression of *pip* or *phzR* rescued phenazine production in *rpeB* mutants whereas multiple copies of *rpeB* or *pip* failed to rescue phenazine production in a *phzR* mutant; for both strains, *phzR* expression restored phenazine production in a *pip* mutant (Wang et al. [Bibr b67][Bibr b68]). Furthermore, expression of *gacS*, *gacA*, and *rpoS* were unchanged in an *rpeB* mutant, and conversely the expression of *rpeA* and *rpeB* were unchanged in an *rpoS* mutant (Wang et al. [Bibr b67][Bibr b68]). These results indicate that RpoS and the RpeB/RpeA independently regulate phenazine production using *pip* as a common regulatory intermediate. Consistent with these findings, data presented here showed that expression of *rpeB* and *rpeA* were not altered by mutation of *gacA*, indicating that RpeB/RpeA and GacS/GacA also do not regulate phenazines in a hierarchic manner. However, the effect of *gacA* mutation on *rpoS* and *pip* expression suggests that GacA influences the expression of additional phenotypes via RpoS and Pip.

### Transcriptomic analyses of genes controlled by Gac: a preliminary survey

#### GacS/GacA functions as a positive regulator of secondary metabolism and exoenzyme production

In 30-84, *gac* mutants are not only deficient in phenazine production, but also HCN, exoprotease, and lipase (Chancey et al. 1999). Consistent with these observations, the transcript abundances of genes that encode for HCN, exoprotease/exopeptidase, and lipase activities were lower in the *gacA* mutant than in WT (Table 2). Additionally, the transcript abundances of 30-84 genes annotated to encode pyrrolnitrin biosynthesis (*prnABCD*) and four genes involved in chitin degradation were lower in the *gacA* mutant than in WT (Table 2). Some of these results were confirmed by qPCR analysis using the *hcnA*, *aprA*, and *prnA* genes (data not shown). These results are consistent with previous findings showing a reduction in the abundance of transcripts of the biosynthetic genes for HCN, pyrrolnitrin, and protease in a *P. protegens* Pf-5 *gacA* mutant (Hassan et al. 2010). In Pf-5, the products of these genes have been demonstrated to be important for biological control activity.

**Table 2:** Mean transcript abundance and ratio of abundances (*ΔgacA*/WT) of genes involved in secondary metabolism and iron uptake in the *gacA* mutant compared with WT strain

Gene ID	Gene	Protein description	Mean RPKM WT	Mean RPKM *ΔgacA*	*ΔgacA*/WT	*P*-value
Hydrogen cyanide						
Pchl3084_2378	*hcnA*	Hydrogen cyanide synthase	746.54	243.92	0.33	0.01
Pchl3084_2379	*hcnB*	Hydrogen cyanide synthase	474.43	70.53	0.15	0.00
Pchl3084_2380	*hcnC*	Hydrogen cyanide synthase	705.07	66.39	0.09	0.00
Protease						
Pchl3084_3127	*aprA*	Metalloprotease	100.83	4.77	0.04	0.02
Pchl3084_2293	*aprX*	Metallopeptidase	164.03	6.44	0.04	0.01
Lipase						
Pchl3084_3120		Lipase	9.4	3.4	0.37	0.00
Pchl3084_3439		Lipase	16.0	4.8	0.3	0.01
Pchl3084_3943		Lipase	25.9	11.4	0.44	0.01
Pchl3084_0883		Phospholipase	50.8	10.6	0.21	0.02
Pyrrolnitrin						
Pchl3084_3143	*prnD*	Aminopyrrolnitrin oxidase	6.94	0.57	0.08	0.04
Pchl3084_3144	*prnC*	Halogenase	12.53	0.85	0.07	0.02
Pchl3084_3145	*prnB*	Pyrrolnitrin biosynthesis	6.88	1.23	0.18	0.00
Pchl3084_3146	*prnA*	Tryptophan halogenase	16.45	1.83	0.11	0.01
Chitinase						
Pchl3084_2020		Chitin-binding domain protein	36.06	4.28	0.12	0.03
Pchl3084_2021		Chitinase	31.17	3.46	0.11	0.01
Pchl3084_3180		Chitinase	57.95	1.83	0.03	0.02
Pchl3084_3181		Chitin-binding domain protein	51.78	3.44	0.07	0.01
Iron uptake						
Pchl3084_3935	*pvdA*	L-ornithine 5-monooxygenase	2.32	5.11	2.20	0.01
Pchl3084_3936	*fpvI*	Sigma-70 factor	12.77	23.78	1.86	0.00
Pchl3084_0320		TonB-dependent outer membrane receptor	1.71	6.19	3.61	0.02
Pchl3084_0855		TonB-dependent outer membrane receptor	23.45	85.19	3.63	0.00
Pchl3084_2518		TonB-dependent outer membrane receptor	2.71	7.17	2.65	0.00
Pchl3084_3174		TonB-dependent outer membrane receptor	8.26	16.77	2.03	0.02
Pchl3084_3659		TonB-dependent outer membrane receptor	3.05	6.43	2.11	0.00
Pchl3084_3848		TonB-dependent outer membrane receptor	7.11	15.08	2.12	0.00
Pchl3084_5801	*ExbB*	TonB system transport protein	55.52	116.65	2.10	0.04

#### Transcripts of genes potentially involved in type VI secretion system, signal transduction, and carbon storage are in lower abundance in the *gacA* mutant relative to WT

Genes annotated as encoding components of a type VI secretion system (T6SS) also were notably underexpressed (2- to ∼100-fold) in the *gacA* mutant relative to WT, suggesting positive control by GacA (Table S4). Again, this is similar to previous findings showing that the expression of Pf-5 genes homologous to the *P. aeruginosa* T6SS (HIS-I type) was reduced in a *gacA* mutant from early to late growth phase (Hassan et al. 2010). In contrast to Pf-5 where all 16 of the differentially expressed genes encoding the T6SS system were located in the same gene cluster, the 24 differentially expressed T6SS genes identified in this study were distributed among six different loci. Previous studies also demonstrated GacS/GacA control of the T6SS in *P. aeruginosa* and *P. syringae* (Silverman et al. 2012). In *P. aeruginosa*, the T6SS delivers bacteriolytic effectors to target cells and is associated with competition among bacterial species (Russell et al. 2011; Julie et al. 2012). Future studies will be required to determine whether 30-84 contains a functional T6SS and if so, its function.

Genes annotated as encoding enzymes involved in polyhydroxyalkanoate (PHA) synthesis and utilization also were underexpressed (2- to ∼100-fold) in the *gacA* mutant relative to WT, suggesting positive control by GacA (Table S5). PHAs are bacterial storage materials produced by many fluorescent pseudomonads including *P. aeruginosa*, *P. putida*, *and P. chlororaphis* (*aureofaciens*) under nutrient conditions in which carbon is in relatively high abundance, but other nutrients are limited (Nishikawa et al. 2002; Pham et al. 2004; Hervas et al. 2008). Genes included *phaD*, a TetR-like transcriptional regulator that in *P. putida* KT2442 activates the *pha* cluster (*phaC1ZC2D*) conferring the ability to degrade PHAs (de Eugenio et al. 2010). In *P. oleovorans* GPo1, *phaD* is transcribed as part of the *pha* operon and controls its own transcription as well as the transcription of the *phaIF* operon, which in turn is involved in the regulation of *pha* genes (Prieto et al. 1999). Consistently, in this study, the transcripts of *phaC1*, *phaZ*, *phaC2*, *phaI*, and *phaF* also were underrepresented (2- to 17-fold) in the *gacA* mutant relative to the WT (Table S5). Previously, it was shown that the PHA synthase *phaC2* is underexpressed in an *rpoS* mutant of *P. chlororaphis* PCL1391 (Girard et al. 2006b). Work with *P. aeruginosa* PAO1 demonstrated that mutants deficient in PHA biosynthesis were less tolerant of heat shock both in liquid culture and in biofilms and were altered in attachment and biofilm architecture relative to WT (Pham et al. 2004). Moreover, comparison of alginate production by PHA-negative derivatives of WT PAO1 and the alginate-overproducing strain, FRD1, led the authors to suggest that the alginate and PHA pathways compete for acyl-CoA precursors (Pham et al. 2004). In 30-84, the transcript abundance of the alginate regulatory gene *algK* was fourfold higher in the *gacA* mutant compared with WT, however, the majority of genes annotated as being involved in alginate synthesis were unchanged. The roles of PHA or alginate production and utilization by 30-84 have yet to be determined, but it is interesting to consider given PHA's potential for influencing bacterial survival and biofilm formation.

Differential transcript abundance of 23 genes annotated as being involved in signal transduction or transcription regulation suggest that these genes also were regulated by GacA (Table S6). Among them, transcripts of 21 genes were underrepresented in the *gacA* mutant compared with WT. Most of these genes are functionally uncharacterized suggesting there remains a large gap in our understanding of *P. chlororaphis* gene regulation by the Gac system.

#### Transcripts of genes involved in iron acquisition are in higher abundance in the *gac* mutant relative to WT: potential contribution to the Gac^−^ phenotype

Hyper-fluorescence is a consistent phenotype of *gac* mutants among fluorescent pseudomonads, including 30-84 (Chancey et al. 2002). It was shown previously that the Gac system negatively controls a large number of genes involved in iron acquisition (Hassan et al., 2010). Transcripts of nine genes annotated to be involved in iron acquisition were two- to threefold more abundant in the *gacA* mutant than the WT (Table 2). Six of them were annotated as TonB-dependent outer membrane receptors potentially involved in the uptake of ferric pyoverdine siderophores. According to current siderophore uptake models, when ferric pyoverdine is recognized by its cognate TonB outer membrane receptor, it is transported into the periplasmic space via a two-gate mechanism (Schalk 2008). TonB and proton motive force provide the energy for siderophore uptake and cause the release of the iron, which is subsequently transported into the cytoplasm through an ABC transporter (Schalk 2008). Consistent with increased iron uptake, transcripts of *pvdA* (involved in fluorescent pyoverdine biosynthesis), *exbB* (a cytoplasmic membrane protein), and *fpvI* (an RNA polymerase sigma-70 factor that recruits RNA polymerase for expression of iron transport genes) were overrepresented in the *gacA* mutant compared with WT. Previously it was shown that insertion of Tn5 into the *pvdA* locus of 30-84 resulted in the loss of fluorescence on low iron media (data not shown), linking fluorescence with pyoverdine synthesis. These results are consistent with overproduction of pyoverdine being partially responsible for the hyper-fluorescent phenotype typical of *gac* mutants of *Pseudomonas* and suggest a possible benefit to having Gac^−^ phenotypes in mixed populations.

#### Transcripts of genes involved in preparation for exponential growth are in higher abundance in the *gac* mutant relative to WT: GacA regulation of sigma factors and genes involved in translation

Protein synthesis is a highly coordinated process that requires ribosomes composed of multiple proteins and ribosomal RNAs, mRNA, tRNAs, initiation and elongation factors, and many other genes. One of the most dramatic transcriptional consequences of loss of *gacA* in *P. chlororaphis* 30-84 was the overexpression (two- to fourfold) relative to the WT of 50 genes involved in translation, including those encoding most of the large and small subunit ribosomal proteins, the translation initiation factor IF-3, and the three translation elongation factors (Table S7). Because the ribosomal protein transcripts were increased in the *gacA* mutant, it is logical to speculate that rRNAs also were increased. However, due to the rRNA depletion steps, the RNA-seq only detected small amounts of rRNA transcripts. Therefore, we performed qPCR to quantify the relative abundance of the 16S rRNA in the *gacA* mutant compared with the WT. Relative to WT, the abundance of 16S rRNA was significantly higher (1.8 ± 0.5-fold) in the *gacA* mutant, suggesting higher expression of ribosomal RNA. In addition, tRNA genes for transferring aspartate, glutamine, lysine, isoleucine, and alanine were overexpressed in the *gacA* mutant compared with WT; whereas threonine and glycine tRNAs were underexpressed (Table S7). A unique consequence of these findings was that due to the consistency of *rpoD* expression relative to 16S rRNA abundance, *rpoD* was used as the reference for all qPCR analyses comparing gene expression in the *gacA* mutant and WT.

In previous study with 30-84 and other pseudomonads, it was found that mutations in *gacA* or *gacS* resulted in earlier transition of cells from lag to exponential phase growth, and this was suggested as an explanation for the competitive advantage of *gac* mutants in liquid broth cultures (Duffy and Defago 2000; Chancey et al. 2002; van den Broek et al. 2005; Driscoll et al., 2011). Although the precise mechanism(s) that facilitate *gac* mutants entering exponential growth phase earlier than the WT is unclear, we speculate that the reduction in *rpoS* expression in *gac* mutants relative to WT may contribute to this effect. Differential regulation of the stationary phase sigma factor RpoS, but not the housekeeping sigma factor RpoD in a *gacA* mutant is particularly interesting given that RpoS competes with RpoD for binding to RNA polymerase core enzymes (Farewell et al. 1998; Battesti et al. 2011). This competition, in part, determines overall patterns of gene expression in the cell. Most research on RpoS has focused on its role in the transition from exponential to stationary phase growth. However, recently Rolfe et al. (2012) showed that during the transition from lag to exponential phase growth *rpoS* expression increased transiently, presumably to enable the cell to respond to oxidative challenges due to the introduction of the stationary phase cells into oxygenated fresh medium. In contrast, levels of RpoD increased twofold during this transition period. A reduction in levels of RpoS in *gacA* mutants would result in higher levels of expression of RpoD-regulated genes, which are involved in primary growth. Increased levels of transcription of translational machinery, including ribosomal proteins, rRNAs, tRNAs, and accessory proteins in the *gac* mutant as reported here also would favor earlier entry into exponential growth.

#### The role of GacA in bacterial motility

The Gac system has been shown to differentially control motility in various bacteria. For example, it positively contributes to motility in *E. coli* K-12 (Wei et al. 2001), *P. protegens* Pf-5 (Hassan et al. 2010), and *P. syringae* pv. *tabaci* (Marutani et al. 2008), but negatively in *P. fluorescens* F113 (Martínez-Granero et al. 2012) and *Erwinia amylovora* Ea1189 (Zhao et al. 2009). In both Pf-5 and *P. syringae* pv *tabaci*, the Gac system positively influences the level of transcription of the flagellar genes (Marutani et al. 2008; Hassan et al. 2010). Consistent with the potential role of GacA in enhanced bacterial motility, colonies of 30-84 *gac* mutants typically appear larger on standard culture plates. Bacterial motility was assessed for strain WT, Δ*gacS*, Δ*gacA*, and 30-84ZN by inoculating bacterial cells onto motility plates (0.25% agar) and measuring the diameter of the area colonized by bacterial cells at 24 and 48 h as described previously (Wang et al. 2011). The Gac mutants exhibited enhanced motility compared with that of the WT strain 12 and 24 h following inoculation (Fig. S1A and B). In contrast, phenazine deficient derivative 30-84ZN *(phzB::lacZ)* exhibited a similar motility pattern to the WT suggesting that the increased motility in the *gac* mutant was not due to lack of phenazine production. Diameters of colonies for the WT, Δ*gacS*, Δ*gacA*, and 30-84ZN at 48 h were about 4.8 ± 0.1, 6.4 ± 0.3, 6.5 ± 0.4, and 4.8 ± 0.3 cm (longest dimension from the inoculation point), respectively. These results indicate that the Gac system negatively regulates bacterial motility in 30-84.

It was demonstrated previously that swimming motility in *P. fluorescens* F113 is under negative control by Gac and that this downregulation occurs through the repression of the flagella master regulatory gene *fleQ* (Martínez-Granero et al. 2012). One of the factors affecting motility is flagella production. Moreover, a *P. chlororaphis* 30-84 *fliM* mutant is nonmotile (L. S. Pierson et al., unpubl. data). However, RNA-seq analyses revealed that the transcript abundance of flagellar genes such as *fliA*, *fliM*, and *fliC* were not significantly altered in the *gacA* mutant (Fig. S1C), and these data were verified by qPCR (Fig. S1D). The results suggest that the impact of GacA on motility may be secondary to flagella structure, possibly related to flagellar rotation. Another possibility is that increased swimming motility is related to enhanced surfactant production, as observed in other bacterial species (Wilf et al., 2012; Daniels et al. 2004). However, known surfactant biosynthetic genes such as the *rhlA* or homologs were not detected in *P. chlororaphis* 30-84 genome.

## Conclusions

The role of the ncRNAs *rsmY* and *rsmZ* in the *Pseudomonas* Gac/Rsm signal transduction pathway was first detected by their binding capacity to the regulatory protein RsmA and subsequently RsmE in *P. fluorescens* CHA0, where their interaction contributed to the regulation of HCN, exoprotease, and 2,4-diacetylphloroglucinol biosynthesis important for the biological control capability of the strain (Heeb et al. 2002; Reimmann et al. 2005). In our study, the functionality of the components of the Gac/Rsm signal transduction pathway in the regulation of phenazines and other genes important for biological control in strain 30-84 was examined. Only *rsmZ* and *rsmE* are linked to phenazine production, however, further RNA–protein binding experiments are needed to verify whether direct interactions between RsmE and the ncRNA *rsmZ* are involved in the regulation of phenazine biosynthesis. Interestingly, in *P. fluorescens* CHA0 *rsmE* expression was shown to be regulated negatively by RsmA (Reimmann et al. 2005). What role RsmA plays in Gac-mediated regulation in 30-84 has yet to be determined. In *P. fluorescens* CHA0 and in *P. aeruginosa*, the regulatory effect of *gacA* was attributed exclusively to the direct control of only two ncRNAs *rsmY* and *rsmZ* (Heeb et al. 2002; Brencic and Lory 2009). In *P. aeruginosa*, single *rsmY* and *rsmZ* mutants synthesize intermediate levels of phenazines, whereas the double *rsmY*/*rsmZ* mutant produces less (Kay et al. 2006). Although constitutive expression of *rsmZ* completely restored phenazine, HCN, and exoprotease production in a 30-84 *gacA* mutant, we cannot exclude the possibility that *rsmY* also partially contributes to the production of these key biological control compounds. Future study will be directed toward identifying the potential roles of the three ncRNAs in strain 30-84.

In most *Pseudomonas* species, phenazine production is controlled by combinations of conserved regulatory systems integrated into sensory networks potentially responsive to environmental, nutritional, population, and metabolic inputs (reviewed in Pierson and Pierson 2010). Transcriptomic analysis revealed that the expression of regulatory genes known to be involved in phenazine production (including *phzR* and *phzI, pip*, and *rpoS)* as well as other genes annotated as being involved in trascriptional regulation were affected by a *gacA* in mutation 30-84. Our genetic analysis demonstrated that whereas a *gacA* mutation could be bypassed by either mutation of *rsmE* or constitutive expression of r*smZ*, it could not be rescued by expression of quorum sensing (e.g., constitutive expression of *phzR* with the addition of AHLs) or other regulatory genes. These data provide evidence that the phenazine biosynthesis genes are direct targets of Gac/Rsm regulation and highlight the strength of gene deletion/overexpression analysis in combination with transcriptomic profiling as an approach to study complex regulatory mechanisms. These results also illustrate the multiple-level regulatory role of the Gac/Rsm system in the control of phenazines and suggest that the far-reaching influences of GacA on the transcriptome are likely mediated through intermediate transcriptional regulatory genes.

Our transcriptional profiling revealed that GacA regulates a wide array of biological functions in *P. chlororaphis* 30-84. In this study, transcriptomic analysis identified 771 genes that were differentially expressed in the GacA mutant compared with WT 30-84, with 551 genes downregulated in the *gacA* mutant under our experimental conditions. Consistent with previous observations that *gac* mutants are phenazine, HCN, exoprotease, and lipase deficient, the transcript abundances of genes that code for these phenotypes were reduced in the *gacA* mutant compared with WT 30-84. Additionally, the transcript abundances of 30-84 genes annotated to encode pyrrolnitrin biosynthesis and chitin degradation also were reduced. These results are consistent with previous findings demonstrating that GacS/GacA functions as a positive regulator of secondary metabolites that contribute to biological control activity in *Pseudomonas*. Other genes positively regulated by GacA in strain 30-84 that could be of potential importance in rhizosphere survival, plant–microbe interaction, and biological control include genes annotated as coding for components of a T6SS. Beyond genes involved in well-defined functional processes, the *gacA* regulon included a gene cluster that is involved in the utilization of polyhydroxyalkanoates, important storage molecules in many pseudomonads. Further studies will be required to determine whether either strain contains a functional T6SS and whether strain 30-84 is capable of utilizing polyhydroxyalkanoates. Future studies also will be directed at how Gac-mediated regulation of these potentially beneficial genes is altered under rhizosphere conditions.

Many *Pseudomonas* species identified for biological control demonstrate phenotypic variation resulting from spontaneous mutation in *gacS* or *gacA*. However, phenotypic variation derived from conserved mutation of specific regulatory genes is not limited to *Pseudomonas*, and has been observed in many Gram^−^ and Gram^+^ species resulting from mutations in *gac* or other regulatory genes (van den Broek et al. 2005). The prevalence of *Pseudomonas gac* mutants in laboratory and natural environments suggests that there is some benefit to the *gac* mutant phenotype and thus selection at some level to maintain phenotypic variation in the species. Previous study with strain 30-84 provides evidence that the presence of the *gac* mutants in WT-Gac mixed populations in biofilms and on plant roots can be beneficial (Chancey et al. 2002; Driscoll et al., 2011). Thus, of particular interest in this study was the identification of genes that are expressed at a higher level in 30-84 *gacA* mutants. Our transcriptomic analysis showed that 220 genes that were overexpressed in the *gacA* mutant under our experimental conditions. The most obvious group, genes involved in iron uptake, may increase iron availability especially in the biofilm context. Increased protein translation is another possible benefit, as the shorter transition to exponential growth for *gac* mutants allows them to quickly establish a population. It is worth noting that 52 upregulated genes in the *gacA* mutant are hypothetical or unknown genes, including the highest upregulated gene (Pchl30-84_3493, 26.2-fold increase) (Table S3). Functional characterizations of these highly expressed genes are currently underway.

## Supporting Information

**Figure S1.** (A) Migration phenotypes of WT, *gac* mutants and 30-84ZN at 48 h after incubation. (B) Comparison of the movement distance of WT, *gac* mutants and 30-84ZN. Cells were inoculated on the surface of a motility plate containing 0.25% agar as described previously (Wang 2011). Plates were incubated at 28°C for 48 h, during which the displacement diameter to the outermost edge of the movement area was measured. The experiment was performed at least three times in triplicate. (C) Expression levels of *fliA*, *fliC*, and *fliM* in WT and a *gacA* mutant. RPKM values representing levels of gene expression were calculated from RNA-seq analysis and log transformed. Data points represent means of three replicates ± standard deviations. (D) Verification of RNA-seq data with qPCR. Bacterial strains were grown overnight in LB broth and reinoculated in 5 mL AB medium + 2% casamino acid. Relative expression of *fliA*, *fliC*, and *fliM* genes, normalized to the expression of *rpoD* gene, was determined by qPCR after ∼18 h growth (OD_600_ at 1.2).

**Table S1.** Bacterial strains and plasmids used in this study.

**Table S2.** Oligonucleotides used for gene cloning and qPCR.

**Table S3.** Differentially expressed genes in the *gacA* mutant compared with the WT strain.

**Table S4.** Mean transcript abundance and ratio of abundances (*Δ**gacA*/WT) of type VI secretion system (T6SS) genes in the *gacA* mutant compared with the WT.

**Table S5.** Mean transcript abundance and ratio of abundances (*Δ**gacA*/WT) of polyhydroxyalkanoate (PHA) biosynthetic genes in the *gacA* mutant compared with the WT.

**Table S6.** Mean transcript abundance and ratio of abundances (*Δ**gacA*/WT) of regulatory genes in the *gacA* mutant compared with the WT.

**Table S7.** Mean transcript abundance and ratio of abundances (*Δ**gacA*/WT) of genes involved in protein metabolism in the *gacA* mutant compared with the WT.
