# Supplementary figures and images for: Roles of the Gac-Rsm pathway in the regulation of phenazine biosynthesis in Pseudomonas chlororaphis 30-84
Source: Microbiologyopen. 2013 Apr 21;2(3):505–24. doi: 10.1002/mbo3.90 (PMC3684763; doi:10.1002/mbo3.90)

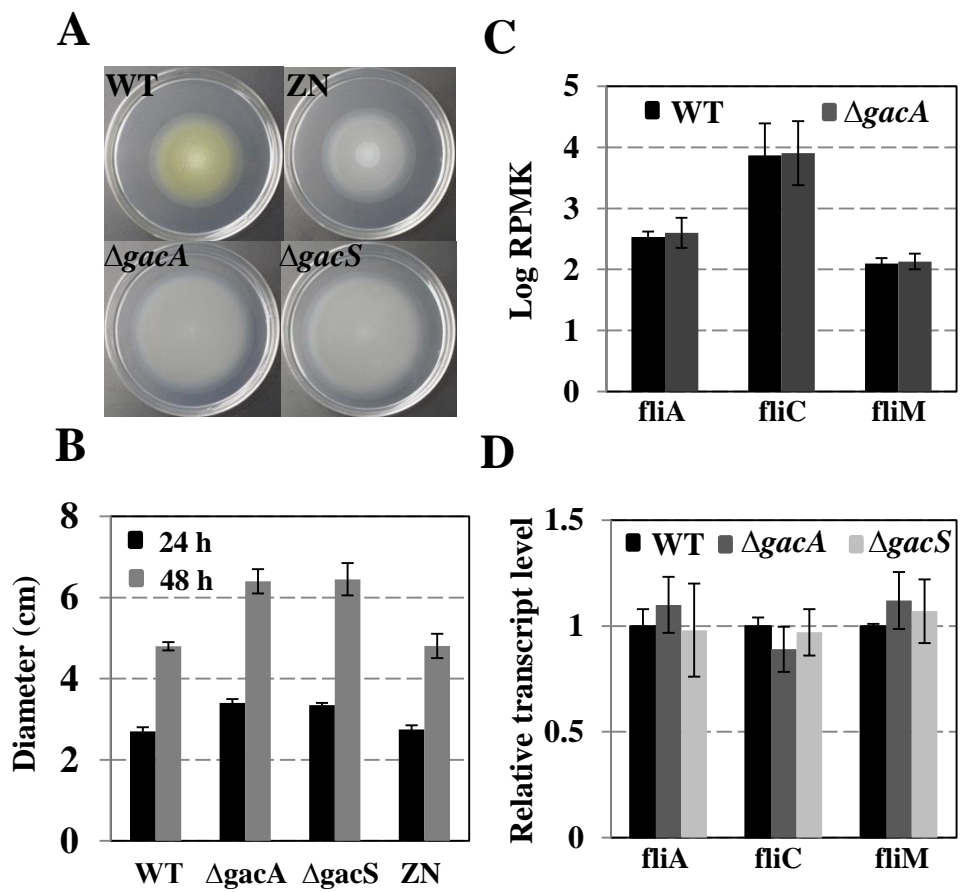

Wang et al., MicrobiologyOpen Supplementary Figure 1

Supplement: Supplementary file 1 [file mbo30002-0505-SD1.pdf]
