# Supplementary material for: Roles of the Gac-Rsm pathway in the regulation of phenazine biosynthesis in Pseudomonas chlororaphis 30-84
Source: Microbiologyopen. 2013 Apr 21;2(3):505–24. doi: 10.1002/mbo3.90 (PMC3684763; doi:10.1002/mbo3.90)
Supplement: Supplementary file 4 [file mbo30002-0505-SD4.docx]

Supplemental Table 1. Bacterial strains and plasmids used in this study.

| Strains and plasmids | Relevant characters | | Reference or source |
| --- | --- | --- | --- |
| ***P. chlororaphis***  30-84 | Phz^+^ Rif^R^ wild-type (WT) | | Whistler and Pierson, 2003 |
| 30-84gacA | Phz^+^ Rif^R^ Km^R^, *gacA*::*km* | | Chancey *et al*., 2003 |
| 30-84gacS | Phz^+^ Rif^R^ Km^R^, *gacS*::*km* | | Chancey *et al*., 2003 |
| 30-84R | Phz^-^ Rif^R^ *phzR*::*lacZ* genomic fusion | | Pierson *et al*., 1994 |
| 30-84I | Phz^-^ Rif^R^ *phzI*::*lacZ* genomic fusion | | Wood and Pierson, 1996 |
| 30-84ZN | Phz^-^ Rif^R^ *phzB*::*lacZ* genomic fusion | | Wood *et al*., 1997 |
| 30-84W | Phz^-^ Rif^R^ spontaneous *gacA* mutant | | Chancey *et al*., 2003 |
| 30-84ZW | Phz^-^ Rif^R^ *phzB*::*lacZ* genomic fusion and spontaneous *gacA* mutant | | Chancey *et al*., 1999 |
| 30-84ZWE | Phz^-^ Rif^R^ *phzB*::*lacZ* genomic fusion and spontaneous *gacA* mutant, *rsmA*::EZ:TN | | This study |
| 30-84Ice | Phz^-^ Rif^R^ *phzB*::*inaZ* genomic fusion | | Wood *et al*., 1997 |
| 30-84IZ | Phz^+^ Rif^r^ *phzB*::*lacZ* and *phzI*::*npt* genomic fusion, Km^R^ | | Wood and Pierson, 1996 |
| ***E. coli*** | | | |
| DH5α | | F^-^ *recA1 endA1 hsdR17 supE44 thi-1 gyrA96 relA1* 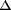(*argF-lacZYA*)*I169* 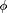80*lacZ*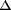M15 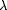^-^ | GIBCO-BRL |
| HB101 | | F^-^ *hsdS20*(r_B_^-^ m_B_^-^) *supE44 recA1 ara14 proA2 lacY1 galK2 rpsL20 xyl-5 mtl-5* 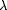^-^ | GIBCO-BRL |
| **Plasmids** | | | |
| pLAFR3 | IncP1 *cos*^+^ *rlx*^+^ Tc^R^ | | Staskawicz *et al*., 1987 |
| pIC20H | ColE1, Ap^R^ | | Marsch *et al*., 1984 |
| pKOK6.1 | Ap^R^, Cm^R^, Km^R^, contains *lacZ*-Km promoter-less cartridge | | **Kokotek and Lotz,** 1989 |
| pPROBE-GT2 | Gn^R^, GFP based promoter trap vector containing a promoter-less *gfp* gene | | Miller *et al*., 2000 |
| pPROBE-KT2 | Km^R^, GFP based promoter trap vector containing a promoter-less *gfp* gene | | Miller *et al*., 2000 |
| pPROBE-KT2lacZ | Km^R^, LacZ based promoter trap vector containing a promoter-less *lacZ* gene | | Wang *et al*., 2012 |
| pLAFR3- P*_tac-_rsmX* | pLAFR3 containing *rsmX* driven by P*_tac_* | | This study |
| pLAFR3- P*_tac-_rsmY* | pLAFR3 containing *rsmY* driven by P*_tac_* | | This study |
| pLAFR3- P*_tac-_rsmZ* | pLAFR3 containing *rsmZ* driven by P*_tac_* | | This study |
| pLAFR3-*rsmA* | pLAFR3 containing 812 bp *rsmA* | | This study |
| pLAFR3-*rsmE* | pLAFR3 containing 700 bp *rsmE* | | This study |
| pGT2-P*_tac_*-*phzR* | pPROBE-GT2 containing *phzR* gene driven by P*_tac_* | | This study |
| pKT2-P*_rsmZ_*-*gfp* | 630-bp DNA fragment containing promoter sequence of *rsmZ* gene in pPROBE-KT2 | | This study |
| pKT2-P*_rsmZ_*-*lacZ-gfp* | pKT2-P*_rsmZ_*-*gfp*, with *lacZ* inserted in front of GFP | | This study |

^a^ Km^R^, Ap^R^, Gn^R^, Cm^R^, Rif^R^ and Tc^R^ = kanamycin, ampicillin, gentamycin, chloramphenicol, rifampin and tetracycline resistance, respectively.

Supplemental Table S2. Oligonucleotides used for gene cloning and qPCR.

| Oligonucleotide^a^ | Sequence (5’-3’) |
| --- | --- |
| rsmX1 | GCCGAATTCGAGCTGTTGACAATTAATCATCGGCTCGTATAATGTGTGGccaactgcagcgcaggacgc(*Eco*RI) |
| rsmX2 | GCGGGATCCctgctcagtggcgaacag (*Bam*HI) |
| rsmY1 | GCCGAATTCGAGCTGTTGACAATTAATCATCGGCTCGTATAATGTGTGGatggacgtcgcgcaggaagc (*Eco*RI) |
| rsmY2 | GCGGGATCCggcagacccacagccatac (*Bam*HI) |
| rsmZ1 | GCCGAATTCGAGCTGTTGACAATTAATCATCGGCTCGTATAATGTGTGGtgtcgacggatagacacagc (*Eco*RI) |
| rsmZ2 | GCGGGATCCagatctggcccaacatcact (*Bam*HI) |
| rsmA1 | CCGGAATTCcacggggcgtatattaacca (*Eco*RI) |
| rsmA2 | CGCGGATCCgtacctggaactggctgtgc (*Bam*HI) |
| rsmE1 | CCGGAATTCCGCCATTGCCATCGCTAC (*Eco*RI) |
| rsmE2 | CGCAAGCTTCGACGTGCTCCTGAACGC (*Hin*dIII) |
| phzR1 | GCCGAATTCGAGCTGTTGACAATTAATCATCGGCTCGTATAATGTGTGGcccactcagcaccgccacgc (*Eco*RI) |
| phzR2 | CGCGGATCCtggcgaagttcaaggtgat (*Bam*HI) |
| gacART1 | GATGACCATGATCTCGTTCG |
| gacART2 | CATCTTGACGTCCATGAGGA |
| gacSRT1 | AAAACCTGGAAACCATCGAG |
| gacSRT2 | GGTGGGTGAAACCGAGAATA |
| rpeBRT1 | CATCCTTCTGGTCGAAGACG |
| rpeBRT2 | AGGTCGAGAATCACCAGGTC |
| phzRRT1 | CGCAAGGATAATCCCATCAG |
| phzRRT2 | CACATTCCCTACCGCTGAAC |
| phzIRT1 | CTACCTCCTGGCGTTCAATG |
| phzIRT2 | GAAGCGAGTCATTTCCCAGA |
| phzYRT1 | CGCCGCCATGAACTATTTAC |
| phzYRT2 | GACGTTGTACCACTCCCAGT |
| pipRT1 | AAAAGACCCGCGAGAACATT |
| pipRT2 | ACGTACAGCTGCTCCTTGCT |
| rsmYRT1 | aggaagcgcaaagcaataac |
| rsmYRT2 | tgcagactgtttccctgaca |
| rpoSRT1 | ATCAGTGGCTTTCCGAATTG |
| rpoSRT2 | GACCTTCGACCTGGATCTGA |
| fliART1 | GCTCGATGAGGTCCGTAAAG |
| fliART2 | CCCAAGGTGTCGTTCAAAAT |
| fliMRT1 | GTCTGGTACAGACCGAACAC |
| fliMRT2 | TGTTGAACATGCTGATACGG |
| fliCRT1 | CTGCAAATCGCTACCCGTAT |
| fliCRT2 | GAACAGCCAGTTCACGCATA |
| rsmART1 | GCGCAGAAAGCCTGATTATT |
| rsmART2 | ATGGCTTGGTTCTTCGTCCT |
| rsmXRT1 | GCGCTCAGGGTCATGGAT |
| rsmXRT2 | GAAGCGGGGTTTCTTCAA |
| rpoDRT1 | ACGTCCTGAGCGGTTACATC |
| rpoDRT2 | CTTTCGGCTTCTTCTTCGTC |
| 16SRT1 | ACGTCCTACGGGAGAAAGC |
| 16SRT2 | CGTGTCTCAGTTCCAGTGTGA |

^a^Underlined nucleotides are restriction sites added and the restriction enzymes are indicated at the end of primers

Supplemental Table S3. Differentially expressed genes in the *gacA* mutant compared to the WT strain. Supplemental Table S4. Mean transcript abundance and ratio of abundances (*∆gacA*/WT) of type VI secretion system (T6SS) genes in the *gacA* mutant compared to the WT.

| **Gene ID** | **Gene** | **Protein description** | **Mean**  **RPKM WT** | **Mean**  **RPKM**  ***∆gacA*** | ***∆gacA*/WT** | **p-value** |
| --- | --- | --- | --- | --- | --- | --- |
| Pchl3084_0231 |  | T6SS Vgr family protein | 206.55 | 3.76 | 0.02 | 0.00 |
| Pchl3084_0232 |  | T6SS effector | 12557.23 | 156.14 | 0.01 | 0.00 |
| Pchl3084_2844 |  | T6SS Vgr family protein | 92.29 | 19.93 | 0.22 | 0.00 |
| Pchl3084_3403 |  | T6SS regulatory | 1.40 | 0.37 | 0.26 | 0.04 |
| Pchl3084_3411 | *tssK1* | T6SS protein | 1.55 | 0.33 | 0.21 | 0.03 |
| Pchl3084_3412 | *tssJ1* | T6SS lipoprotein | 4.23 | 0.22 | 0.05 | 0.02 |
| Pchl3084_3420 | *tssE1* | T6SS protein | 4.14 | 1.11 | 0.27 | 0.00 |
| Pchl3084_3442 |  | T6SS Vgr family protein | 3.81 | 1.50 | 0.39 | 0.01 |
| Pchl3084_3989 |  | T6SS Vgr family protein | 2.19 | 0.67 | 0.31 | 0.00 |
| Pchl3084_5816 |  | T6SS Vgr family protein | 31.61 | 3.13 | 0.10 | 0.01 |
| Pchl3084_5819 |  | T6SS Vgr family protein | 124.13 | 10.47 | 0.08 | 0.01 |
| Pchl3084_5822 | *tssM2* | T6SS protein | 307.57 | 18.40 | 0.06 | 0.00 |
| Pchl3084_5823 | *tssL2* | T6SS protein | 304.92 | 18.92 | 0.06 | 0.00 |
| Pchl3084_5824 | *tssK2* | T6SS protein | 325.19 | 27.20 | 0.08 | 0.00 |
| Pchl3084_5825 | *tssJ2* | T6SS lipoprotein | 325.18 | 22.97 | 0.07 | 0.00 |
| Pchl3084_5826 | *fha2* | T6SS protein | 252.57 | 23.45 | 0.09 | 0.00 |
| Pchl3084_5829 | *clpV2* | T6SS ATPase | 186.83 | 11.94 | 0.06 | 0.00 |
| Pchl3084_5830 | *tssG2* | T6SS protein | 142.04 | 15.70 | 0.11 | 0.00 |
| Pchl3084_5831 | *tssF2* | T6SS protein | 192.91 | 27.99 | 0.15 | 0.01 |
| Pchl3084_5836 |  | T6SS system lysozyme-related protein | 649.67 | 35.65 | 0.05 | 0.00 |
| Pchl3084_5837 |  | T6SS protein, EvpB/family | 3584.80 | 146.72 | 0.04 | 0.00 |
| Pchl3084_5838 |  | T6SS protein | 3771.34 | 164.56 | 0.04 | 0.00 |
| Pchl3084_5839 |  | T6SS protein | 263.58 | 40.74 | 0.15 | 0.00 |
| Pchl3084_3901 | *vgrG* | type VI effector protein | 186.83 | 20.17 | 0.11 | 0.00 |

Supplemental Table S5. Mean transcript abundance and ratio of abundances (*∆gacA*/WT) of polyhydroxyalkanoate (PHA) biosynthetic genes in the *gacA* mutant compared to the WT.

| **Gene ID** | **Gene** | **Protein description** | **Mean**  **RPKM WT** | **Mean**  **RPKM**  ***∆gacA*** | ***∆gacA*/WT** | **p-value** |
| --- | --- | --- | --- | --- | --- | --- |
| Pchl3084_0428 |  | polyhydroxyalkanoic acid system protein | 92.74 | 75.58 | 0.81 | 0.02 |
| Pchl3084_0429 | phaI | poly(3-hydroxyalkanoate) granule-associated protein | 147.74 | 66.83 | 0.45 | 0.03 |
| Pchl3084_0430 | phaF | poly(3-hydroxyalkanoate) granule-associated protein | 322.04 | 100.29 | 0.31 | 0.01 |
| Pchl3084_0431 | phaD | transcriptional regulator | 23.97 | 2.18 | 0.09 | 0.01 |
| Pchl3084_0432 | phaC | poly(3-hydroxyalkanoate) synthase 2 | 64.69 | 4.16 | 0.06 | 0.02 |
| Pchl3084_0433 | phaZ | poly(3-hydroxyalkanoate) depolymerase | 62.70 | 19.01 | 0.30 | 0.02 |
| Pchl3084_0434 | phaA | poly(3-hydroxyalkanoate) synthase 1 | 111.18 | 32.73 | 0.29 | 0.02 |

Supplemental Table S6. Mean transcript abundance and ratio of abundances (*∆gacA*/WT) of regulatory genes in the *gacA* mutant compared to the WT.

| **Gene ID** | **Gene** | **Protein description** | **Mean**  **RPKM WT** | **Mean**  **RPKM**  ***∆gacA*** | ***∆gacA*/WT** | **p-value** |
| --- | --- | --- | --- | --- | --- | --- |
| Pchl3084_5828 |  | sigma-54 dependent transcriptional regulator | 154.10 | 10.61 | 0.07 | 0.00 |
| Pchl3084_0431 | PhaD | transcriptional regulator | 23.97 | 2.18 | 0.09 | 0.01 |
| Pchl3084_1034 | YgiT | transcriptional regulator | 30.38 | 10.35 | 0.34 | 0.00 |
| Pchl3084_0750 |  | transcriptional regulator, AraC family | 8.15 | 3.62 | 0.44 | 0.00 |
| Pchl3084_2523 |  | transcriptional regulator, AraC family | 11.79 | 4.34 | 0.37 | 0.02 |
| Pchl3084_3104 |  | transcriptional regulator, AraC family | 6.42 | 2.74 | 0.43 | 0.03 |
| Pchl3084_3596 |  | transcriptional regulator, AraC family | 6.21 | 2.16 | 0.35 | 0.03 |
| Pchl3084_5873 |  | transcriptional regulator, AraC family | 1.66 | 4.24 | 2.55 | 0.03 |
| Pchl3084_3067 |  | transcriptional regulator, AsnC family | 2.79 | 1.27 | 0.46 | 0.00 |
| Pchl3084_2910 |  | transcriptional regulator, Crp/Fnr family | 4.76 | 2.09 | 0.44 | 0.02 |
| Pchl3084_2222 |  | transcriptional regulator, GntR family | 15.28 | 3.45 | 0.23 | 0.01 |
| Pchl3084_4165 |  | transcriptional regulator, GntR family | 4.92 | 1.64 | 0.33 | 0.04 |
| Pchl3084_4994 |  | transcriptional regulator, GntR family | 13.48 | 4.23 | 0.31 | 0.01 |
| Pchl3084_2728 |  | transcriptional regulator, GntR family/aminotransferase | 358.52 | 16.33 | 0.05 | 0.00 |
| Pchl3084_0458 |  | transcriptional regulator, LysR family | 9.51 | 4.36 | 0.46 | 0.03 |
| Pchl3084_3436 |  | transcriptional regulator, LysR family | 2.61 | 0.73 | 0.28 | 0.01 |
| Pchl3084_3455 |  | transcriptional regulator, LysR family | 6.72 | 3.20 | 0.48 | 0.00 |
| Pchl3084_3330 |  | sensor histidine kinase, BaeS family | 6.71 | 3.12 | 0.47 | 0.01 |
| Pchl3084_3908 |  | sensor histidine kinase/response regulator | 9.16 | 4.42 | 0.48 | 0.01 |
| Pchl3084_5555 | ChpA | sensor histidine kinase/response regulator | 39.10 | 16.87 | 0.43 | 0.00 |
| Pchl3084_4980 |  | sensory box methyl-accepting chemotaxis protein | 1.19 | 2.58 | 2.17 | 0.05 |
| Pchl3084_2371 |  | sensory box transcriptional regulator | 3.99 | 40.7 | 0.38 | 0.02 |
| Pchl3084_1883 |  | sensory box-containing diguanylate cyclase | 118.75 | 20.17 | 0.38 | 0.00 |

Supplemental Table S7. Mean transcript abundance and ratio of abundances (*∆gacA*/WT) of genes involved in protein metabolism in the *gacA* mutant compared to the WT.

| **Gene ID** | **Protein description** | **Mean**  **RPKM WT** | **Mean**  **RPKM**  ***∆gacA*** | ***∆gacA*/WT** | **p-value** |
| --- | --- | --- | --- | --- | --- |
| **Ribosomal protein** |  |  |  |  |  |
| Pchl3084_1556 | ribosomal large subunit protein | 67.8 | 189.7 | 2.8 | 0.00 |
| Pchl3084_5331 | ribosomal protein L1 | 2350.44 | 6012.02 | 2.56 | 0.00 |
| Pchl3084_5330 | ribosomal protein L10 | 4327.11 | 10827.99 | 2.5 | 0.00 |
| Pchl3084_5332 | ribosomal protein L11 | 2951.72 | 7446.45 | 2.52 | 0.00 |
| Pchl3084_4866 | ribosomal protein L13 | 3378.01 | 7089.68 | 2.1 | 0.00 |
| Pchl3084_5310 | ribosomal protein L14 | 2462.47 | 7103.67 | 2.88 | 0.00 |
| Pchl3084_5301 | ribosomal protein L15 | 3140.89 | 7139.84 | 2.27 | 0.00 |
| Pchl3084_5313 | ribosomal protein L16 | 2108.93 | 5697.95 | 2.7 | 0.00 |
| Pchl3084_5294 | ribosomal protein L17 | 2982.19 | 6522.94 | 2.19 | 0.00 |
| Pchl3084_5304 | ribosomal protein L18 | 2827.32 | 5909.62 | 2.09 | 0.01 |
| Pchl3084_1080 | ribosomal protein L19 | 2132.72 | 4316.41 | 2.02 | 0.01 |
| Pchl3084_5317 | ribosomal protein L2 | 2362.77 | 5392.91 | 2.28 | 0.00 |
| Pchl3084_2045 | ribosomal protein L20 | 2150.63 | 5961.55 | 2.77 | 0.00 |
| Pchl3084_5109 | ribosomal protein L21 | 2322.78 | 5604.94 | 2.41 | 0.00 |
| Pchl3084_5315 | ribosomal protein L22 | 2387.55 | 5189 | 2.17 | 0.01 |
| Pchl3084_5318 | ribosomal protein L23 | 2154.57 | 4915.36 | 2.28 | 0.01 |
| Pchl3084_5309 | ribosomal protein L24 | 2653.09 | 6401.9 | 2.41 | 0.00 |
| Pchl3084_4928 | ribosomal protein L25 | 2093.41 | 4739.93 | 2.26 | 0.00 |
| Pchl3084_5096 | ribosomal protein L27 | 2017.72 | 4548.54 | 2.25 | 0.01 |
| Pchl3084_5783 | ribosomal protein L28 | 3442.27 | 7062.08 | 2.05 | 0.00 |
| Pchl3084_5312 | ribosomal protein L29 | 1620.34 | 4452.05 | 2.75 | 0.00 |
| Pchl3084_5320 | ribosomal protein L3 | 2423.92 | 6108.8 | 2.52 | 0.00 |
| Pchl3084_5302 | ribosomal protein L30 | 3930.14 | 8005.74 | 2.04 | 0.01 |
| Pchl3084_0441 | ribosomal protein L31 | 1404.88 | 3215.88 | 2.29 | 0.00 |
| Pchl3084_1765 | ribosomal protein L32 | 1361.81 | 2954.65 | 2.17 | 0.03 |
| Pchl3084_5782 | ribosomal protein L33 | 2547.77 | 6091.8 | 2.39 | 0.04 |
| Pchl3084_2044 | ribosomal protein L35 | 3525.38 | 9229.27 | 2.62 | 0.00 |
| Pchl3084_5299 | ribosomal protein L36 | 1945.31 | 4274.71 | 2.2 | 0.01 |
| Pchl3084_5319 | ribosomal protein L4 | 2729.77 | 6837.2 | 2.5 | 0.00 |
| Pchl3084_5308 | ribosomal protein L5 | 3277.65 | 7987.55 | 2.44 | 0.00 |
| Pchl3084_5305 | ribosomal protein L6 | 3631.06 | 8269.96 | 2.28 | 0.01 |
| Pchl3084_5329 | ribosomal protein L7 | 3803.1 | 9568.1 | 2.52 | 0.01 |
| Pchl3084_0570 | ribosomal protein L9 | 2132.25 | 5722.87 | 2.68 | 0.00 |
| Pchl3084_4189 | ribosomal protein S1 | 3009.31 | 6389.4 | 2.12 | 0.00 |
| Pchl3084_5321 | ribosomal protein S10 | 3752.91 | 8575.37 | 2.28 | 0.00 |
| Pchl3084_5325 | ribosomal protein S12 | 2770.23 | 7079.12 | 2.56 | 0.02 |
| Pchl3084_5307 | ribosomal protein S14 | 2751.36 | 6479.57 | 2.36 | 0.00 |
| Pchl3084_0838 | ribosomal protein S15 | 3027.99 | 6133.39 | 2.03 | 0.00 |
| Pchl3084_1077 | ribosomal protein S16 | 1874.99 | 4779.24 | 2.55 | 0.00 |
| Pchl3084_0568 | ribosomal protein S18 | 1950.38 | 6370.78 | 3.27 | 0.00 |
| Pchl3084_5316 | ribosomal protein S19 | 1916.77 | 4992.27 | 2.6 | 0.00 |
| Pchl3084_1160 | ribosomal protein S2 | 2580.28 | 5686.56 | 2.2 | 0.00 |
| Pchl3084_5396 | ribosomal protein S21 | 1422.35 | 2993.66 | 2.1 | 0.03 |
| Pchl3084_5314 | ribosomal protein S3 | 3338.06 | 8845.21 | 2.65 | 0.00 |
| Pchl3084_5303 | ribosomal protein S5 | 3549.44 | 7940.31 | 2.24 | 0.00 |
| Pchl3084_0567 | ribosomal protein S6 | 3566 | 8556.41 | 2.4 | 0.00 |
| Pchl3084_5324 | ribosomal protein S7 | 2111.81 | 5317.08 | 2.52 | 0.01 |
| Pchl3084_5306 | ribosomal protein S8 | 3763.6 | 8113.64 | 2.16 | 0.00 |
| **Translation** |  |  |  |  |  |
| Pchl3084_1160 | translation elongation factor Ts | 3186.52 | 6797.31 | 2.13 | 0.00 |
| Pchl3084_5322 | translation elongation factor Tu | 1505.75 | 3651.13 | 2.42 | 0.01 |
| Pchl3084_2043 | translation initiation factor IF-3 | 5576.94 | 11293.27 | 2.02 | 0.01 |
| Pchl3084_4387 | translational regulator RsmA | 1963.74 | 4313.13 | 2.20 | 0.00 |
| Pchl3084_4929 | peptidyl-tRNA hydrolase | 202.08 | 458.47 | 2.27 | 0.03 |
| **tRNA** |  |  |  |  |  |
| Pchl3084_3982 | tRNA | 38.16 | 0.00 | 0.00 | 0.00 |
| Pchl3084_3984 | tRNA | 58.16 | 0.00 | 0.00 | 0.03 |
| Pchl3084_1724 | tRNA-Gly | 2467.47 | 971.10 | 0.39 | 0.02 |
| Pchl3084_1726 | tRNA-Gly | 2515.51 | 993.16 | 0.39 | 0.02 |
| Pchl3084_5150 | tRNA-methyltransferase | 60.42 | 126.88 | 2.10 | 0.00 |
| Pchl3084_2009 | tRNA-Asp | 66.89 | 140.50 | 2.10 | 0.02 |
| Pchl3084_4926 | tRNA-Gln | 226.05 | 480.69 | 2.13 | 0.04 |
| Pchl3084_2456 | tRNA-hydroxylase | 17.77 | 38.18 | 2.15 | 0.01 |
| Pchl3084_1079 | tRNA-methyltransferase | 1815.80 | 3917.95 | 2.16 | 0.02 |
| Pchl3084_4719 | tRNA-methyltransferase CmoA | 26.19 | 59.06 | 2.25 | 0.01 |
| Pchl3084_4545 | tRNA-Lys | 19.43 | 69.50 | 3.58 | 0.00 |
| Pchl3084_5347 | tRNA-Ile | 6.87 | 35.35 | 5.15 | 0.03 |
| Pchl3084_5346 | tRNA-Ala | 12.07 | 159.23 | 13.20 | 0.01 |
| Pchl3084_0840 | tRNA-Thr | 3078.81 | 1274.79 | 0.41 | 0.02 |
